# Colorectal cancer cells require glycogen synthase kinase-3β for sustaining mitosis via translocated promoter region (TPR)-dynein interaction

**DOI:** 10.18632/oncotarget.24344

**Published:** 2018-01-30

**Authors:** Firli R.P. Dewi, Takahiro Domoto, Masaharu Hazawa, Akiko Kobayashi, Takayuki Douwaki, Toshinari Minamoto, Richard W. Wong

**Affiliations:** ^1^ Faculty of Natural System, Institute of Natural Science and Technology, Kanazawa University, Kanazawa, Japan; ^2^ Division of Translational and Clinical Oncology, Cancer Research Institute, Kanazawa University, Kanazawa, Japan; ^3^ Cell-Bionomics Research Unit, Innovative Integrated Bio-Research Core, Institute for Frontier Science Initiative, Kanazawa University, Kanazawa, Japan; ^4^ WPI Nano Life Science Institute (WPI-NanoLSI), Kanazawa University, Kakuma-machi, Kanazawa, Japan

**Keywords:** cell cycle, colon cancer, dynein, glycogen synthase kinase-3β, translocated promoter region (TPR)

## Abstract

Glycogen synthase kinase (GSK) 3β, which mediates fundamental cellular signaling pathways, has emerged as a potential therapeutic target for many types of cancer including colorectal cancer (CRC). During mitosis, GSK3β localizes in mitotic spindles and centrosomes, however its function is largely unknown. We previously demonstrated that translocated promoter region (TPR, a nuclear pore component) and dynein (a molecular motor) cooperatively contribute to mitotic spindle formation. Such knowledge encouraged us to investigate putative functional interactions among GSK3β, TPR, and dynein in the mitotic machinery of CRC cells. Here, we show that inhibition of GSK3β attenuated proliferation, induced cell cycle arrest at G2/M phase, and increased apoptosis of CRC cells. Morphologically, GSK3β inhibition disrupted chromosome segregation, mitotic spindle assembly, and centrosome maturation during mitosis, ultimately resulting in mitotic cell death. These changes in CRC cells were associated with decreased expression of TPR and dynein, as well as disruption of their functional colocalization with GSK3β in mitotic spindles and centrosomes. Clinically, we showed that *TPR* expression was increased in CRC databases and primary tumors of CRC patients. Furthermore, TPR expression in SW480 cells xenografted into mice was reduced following treatment with GSK3β inhibitors. Together, these results indicate that GSK3β sustains steady mitotic processes for proliferation of CRC cells via interaction with TPR and dynein, thereby suggesting that the therapeutic effect of GSK3β inhibition depends on induction of mitotic catastrophe in CRC cells.

## INTRODUCTION

Colorectal cancer (CRC) is the third most common cancer worldwide, causing over 600,000 deaths per year despite recently declining rates for incidence and mortality [1, 2]. A large number of CRC patients are susceptible to recurrent and metastatic tumors following curative surgery, and many undergo adjuvant systemic therapies including 5-fluorouracil–based chemotherapy. Identification of new therapeutic targets is therefore required to decrease CRC-related mortality. Emerging potential targets are molecules that act as biological machinery in mitosis, a fundamental prerequisite for cancer cell propagation [3–5].

Glycogen synthase kinase-3β (GSK3β), a serine/threonine protein kinase regulating multiple cell signaling pathways, is constitutively active in normal cells. Its activity is finely controlled by the differential phosphorylation of its serine 9 (S9) residue (pGSK3β^S9^, inactive state) and tyrosine 216 (Y216) residue (pGSK3β^Y216^, active state). Depending on its respective primary functions, GSK3β has been implicated in glucose intolerance, neurodegenerative disorders, chronic inflammatory and immunological diseases, and cancer [6–9]; although the various roles described for GSK3β in cancer remain complex and controversial [reviewed in 8]. Notably, roles for GSK3β and microRNAs in epithelial–mesenchymal transition and cancer stem cells were reported recently [10]. Our previous studies found that increased expression of total GSK3β and its active fraction (pGSK3β^Y216^) is a distinct feature of many cancer types [reviewed in 7, 9, 11] including CRC [12–15]. Moreover, we and other groups have demonstrated the preferential therapeutic effect of GSK3β inhibition against these cancers, underscoring this kinase as a promising target in cancer treatment [7–9].

During the multistep process of mitosis, GSK3β localizes to the spindle apparatus and centrosomes [16, 17]. GSK3β phosphorylates and alters the motility of dynein [18], a microtubule minus end-directed molecular motor involved in positioning of the mitotic spindle and microtubule-organizing centers [19–21]. GSK3β also stabilizes bicaudal-D and dynein-interacting complex leading to microtubule anchorage at centrosomes [22]. A recent study reported the therapeutic effect of GSK3β inhibition on cancer via disruption of centrosome homeostasis resulting in mitotic catastrophe [23]. However, little is known about whether and how GSK3β mediates mechanistic processes in the spindle apparatus and centrosomes during mitosis in cancer cells.

The nuclear pore complex (NPC), a cylindrical and symmetric microstructure composed of multiple copies of up to 30 different proteins termed nucleoporins (Nups) (Supplementary Figure 1), is the sole gateway for nucleocytoplasmic exchange of macromolecules [24–26]. Nups are organized into biochemically stable sub-complexes that serve as NPC building blocks [27–29]. From the pore center region, a group of Nups (called FG Nups for their rich content in phenylalanine-glycine repeats) extend and retract their intrinsically disordered FG repeats to form the permeability barrier that bestows selectivity and specificity to the NPC [30]. Indeed, using high-speed atomic force microscopy (HS-AFM), we recently revealed that the native nuclear pore inner channel resembles broken cobwebs or brushes in HCT116 colon cancer cells [31]. Aside from their functions as NPC constituents, we and others also found that several Nups participate in cell division and maintain centrosomal homeostasis [32, 33]. These studies provide new insight into the putative role of certain Nups in the process of cancer cell mitosis [25, 34]. Recent evidence suggests that the nuclear envelope and nuclear transport machinery have emerged as a therapeutic target in oncology to restore physiological homeostasis between the nucleus and cytoplasm [35]. Translocated promoter region (TPR), which normally localizes to the nuclear pore basket, is a potential proto-oncogenic Nup [36–39] (Supplementary Figure 1). The N terminus of TPR undergoes frequent rearrangement with Met, Trk, and Raf oncogenes in gastric and thyroid cancers, resulting in hyperactive tyrosine kinase fusions [40]. TPR comprises a region near the C terminus that is highly enriched in aspartic and glutamic acids, as is observed in many histone chaperones. Moreover, a proteomic analysis of nascent chromatin structures identified TPR as a chromatin-associated protein [41, 42].

We reported that TPR facilitates the biodynamic process of mitosis by translocalizing to the spindle apparatus and centrosomes, whereby it binds and interacts with the MAD1-MAD2 cell cycle checkpoint protein complex and dynein/dynactin molecular motor complex [43, 44]. Moreover, GSK3β could phosphorylate TPR at S2059 *in silico* [45]. Considering all of this background knowledge collectively, we hypothesize that GSK3β may sustain the mitotic process in cancer cells by interacting with critical mitotic mediators such as TPR and dynein sub-complexes.

## RESULTS

### GSK3β inhibition attenuated survival and proliferation of CRC cells

To ascertain the role of GSK3β in tumor cell biology, we examined the effect of GSK3β inhibition on survival and proliferation of CRC cells. Consistent with our previous studies [12–15], GSK3β-specific small-molecule inhibitors AR-A014418 [46] and SB-216763 [47] reduced the proliferation of CRC cells (HCT116, SW480, LoVo, and HT-29) compared with the same cells treated with dimethyl sulfoxide (DMSO, diluent for inhibitors) (Supplementary Figure 2A). This effect was time- and dosage-dependent within the reported pharmacological dosage ranges of respective inhibitors [46, 47]. The same effect was observed in these cancer cell lines following depletion of GSK3β expression by treatment with a specific small interfering RNA (siRNA), whereby depletion efficiency was confirmed by immunoblotting (Supplementary Figure 2B). The effect of GSK3β-specific siRNA was compromised by co-transfection of the constitutively active mutant form of GSK3β (GSK3β S9F-HA; Supplementary Figure 2C). These results reconfirmed that CRC cells depend on GSK3β expression and activity for proliferation.

**Figure 1:**
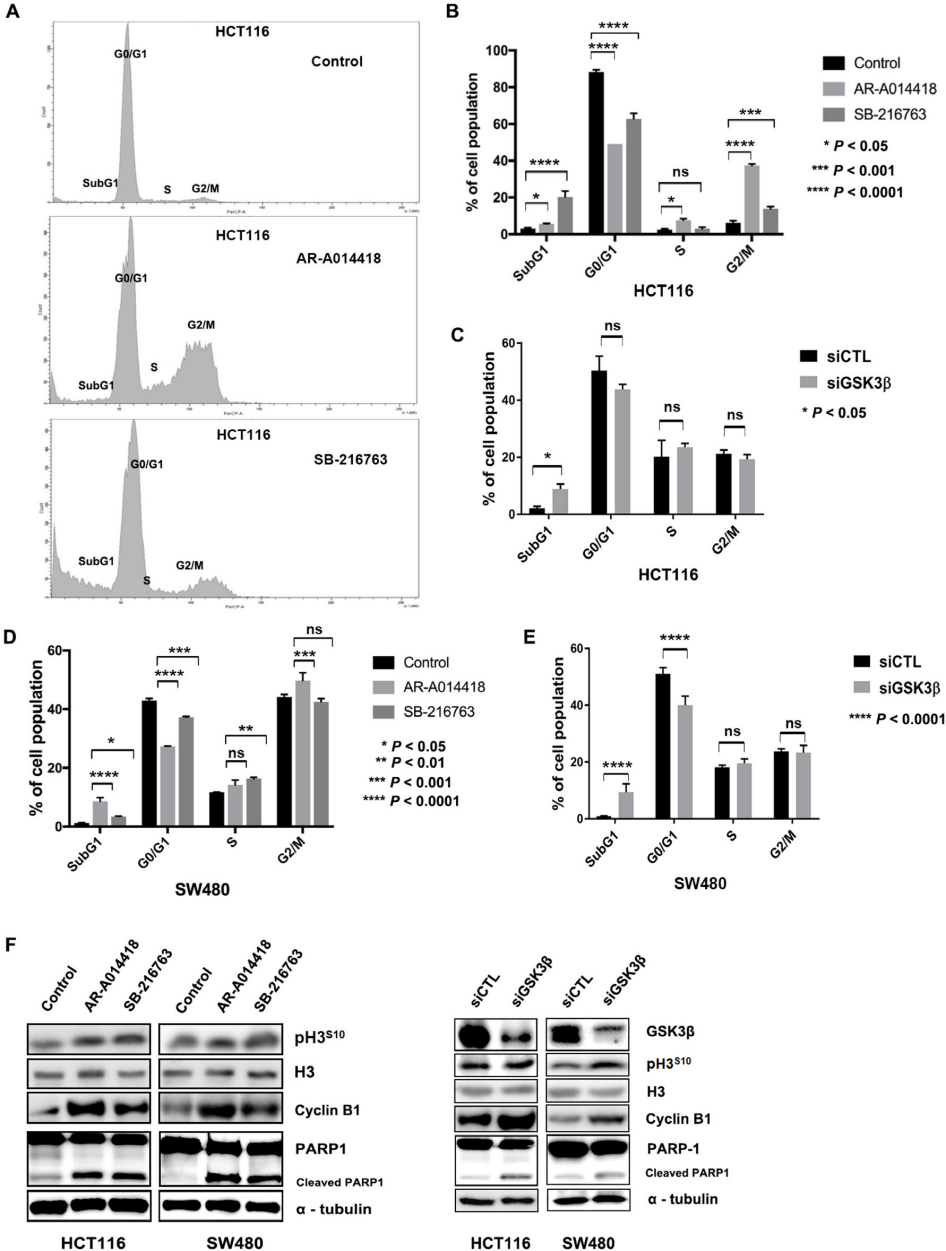
GSK3β inhibition altered cell cycle profile and induced apoptosis (**A**) Changes in cell cycle fractions of HCT116 cells after treatment with DMSO (control), 25 µM AR-A014418, or 25 µM SB-216763 for 96 hours. (**B**) Comparison of DNA histograms for each cell cycle fraction of HCT116 cells after treatment with DMSO (control), AR-A014418, or SB-216763, and (**C**) after treatment with non-specific (siCTL) or GSK3β-specific siRNA (siGSK3β). (**D**) Comparison of DNA histograms for cell cycle fractions of SW480 cells after treatment with DMSO, AR-A014418, or SB-216763, and (**E**) after treatment with siCTL or siGSK3β. Data indicate means ± SD of three separate experiments. *P* value < 0.05, statistically significant difference between cells treated with DMSO and either AR-A014418 or SB-216763. (**F**) Western blotting analysis for expression of cyclin-B1, histone H3, PARP1 and its cleaved fraction, and phosphorylation of histone H3 S10 residue (p-H3^S10^) in HCT116 and SW480 colon cancer cells treated with DMSO (control), AR-A014418, or SB-216763, and after treatment with siCTL or siGSK3β.

Next, we examined whether GSK3β inhibition alters the respective cell cycle fractions in CRC cells. Figure 1A shows a representative DNA histogram of HCT116 cells after treatment with DMSO, AR-A014418, or SB-216763. Analysis by flow cytometry showed that treatment of cells with pharmacological GSK3β inhibitors at 25 µM increased S-phase, G2/M-phase, and sub-G1 fractions, while decreasing the G0/G1-phase fraction in HCT116 (Figure 1B) and SW480 cells (Figure 1D). The same effect was observed following depletion of GSK3β expression in HCT116 (Figure 1C) and SW480 cells (Figure 1E). The results indicated that GSK3β inhibition induced cell cycle arrest at S or G2/M phase, and apoptosis. This effect was associated with increased levels of cyclin-B1 expression and phosphorylation of the S10 residue of histone H3 (p-H3^S10^), which are involved in the G2/M phase transition, and cleaved poly [ADP-ribose] polymerase 1 (PARP-1), a surrogate marker for apoptosis (Figure 1F). Taken together, GSK3β inhibition attenuated cell survival and proliferation by inducing cell cycle arrest and apoptosis in CRC cells.

### GSK3β colocalizes and interacts with TPR and dynein in the centrosome of CRC cells

The cell cycle arrest induced in cancer cells by GSK3β inhibition as shown above (Figure 1) suggests a mechanistic role of this kinase in the biodynamic process of mitosis. We therefore examined the effects of GSK3β inhibition on chromosome segregation and centrosome duplication, two critical mechanistic events during mitosis [3, 4].

**Figure 2:**
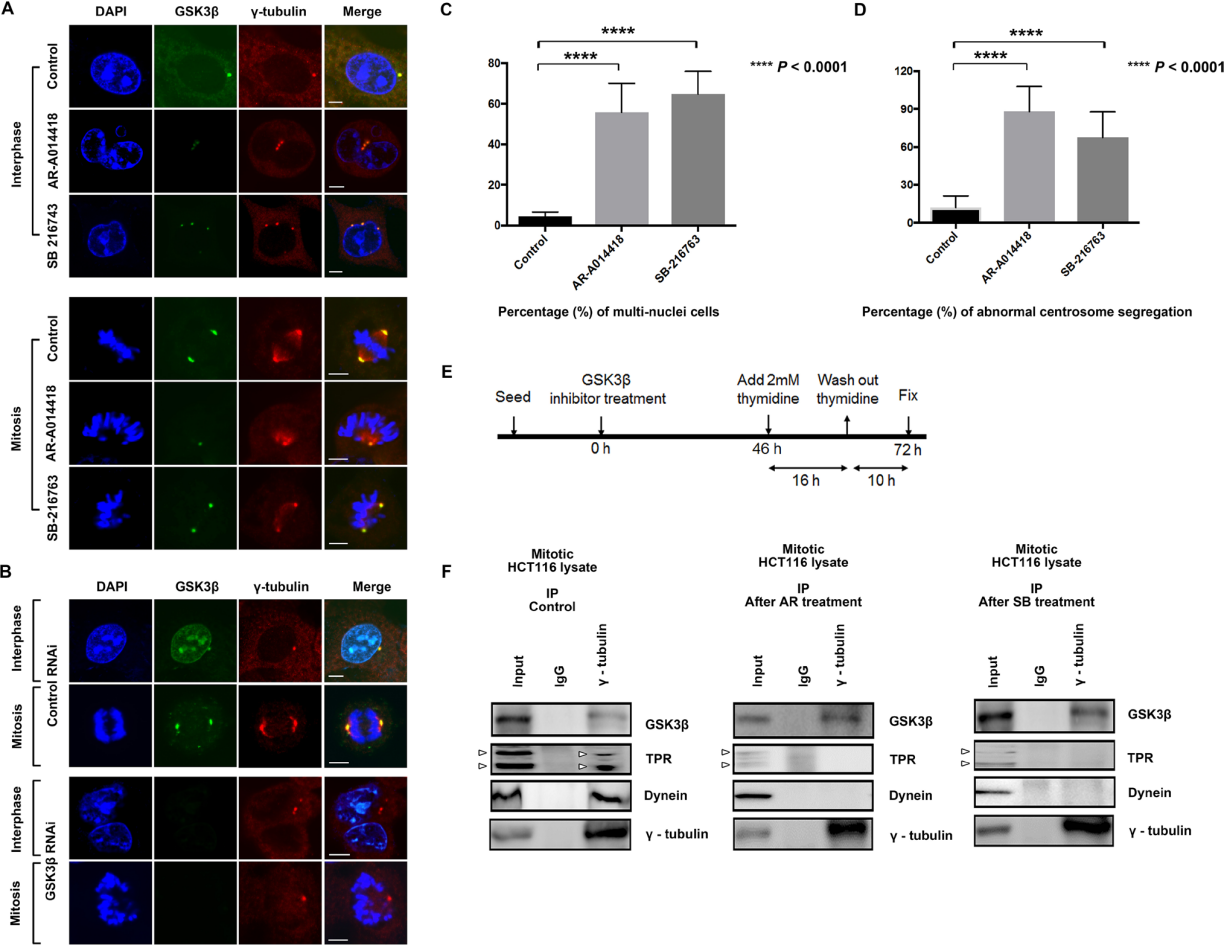
Induction of centrosome abnormalities by GSK3β inhibition (**A**) HCT116 cells were treated with DMSO (control), or 25 μM AR-A014418 or SB-216763 for 72 hours. Cells were immunostained with anti-GSK3β (green) and anti-γ-tubulin (red) antibodies, and nuclei (chromosomes) were counterstained with DAPI (blue) (interphase, upper 12 panels; mitosis, lower 12 panels). Cells with more than two centrosomes or with morphologically abnormal centrosomes after treatment with GSK3β inhibitors are shown. (**B**) Confocal microscopic images of mitotic HCT116 cells transfected with control or GSK3β-specific siRNA for 72 hours. Cells were stained with anti-GSK3β (green) and anti-γ-tubulin (red) antibodies and counterstained with DAPI (blue). Scale bar = 5 μm. Quantification (relative%) of (**C**) multi-nuclear cells (nucleus more than 1) and (**D**) chromosome segregation defects with aberrant centrosomes in cells treated with DMSO (control), 25 μM AR-A014418, or 25 μM SB-216763. Values are based on three independent experiments, counting 100 mitotic cells in each experiment. Mean values with SD are shown. ^****^P value < 0.0001, statistically significant difference between cells treated with DMSO and either of AR-A014418 or SB-216763. (**E**) Protocol for collecting mitotic HCT116 cells after treatment with GSK3β inhibitor. (**F**) Effects of GSK3β inhibition on associations among γ-tubulin (centrosome marker), GSK3β, TPR and dynein (DIC 74.1) in HCT116 cells in mitotic phase. HCT116 cells were treated with DMSO (control) and either AR-A014418 or SB-216763 and synchronized at mitotic phase. Immunoprecipitates (IP) from extracts of mitotic HCT116 cells with anti-γ-tubulin or nonspecific rabbit IgG were analyzed by immunoblotting with indicated antibodies (5% protein extracts were used for input), arrowheads indicate TPR and its isoform.

Here, we visualized centrosomes by immuno-fluorescence labeling of γ-tubulin, a well-known centrosome marker, in HCT116 (Figure 2A) and SW480 cells (Supplementary Figure 3). We found cells with abnormal centrosome number or with abnormal spindles after treatment with GSK3β inhibitor (Figure 2A, and Supplementary Figure 3) or GSK3β RNAi (Figure 2B) for 72 h. Treatment with either AR-A014418 or SB-216763 significantly increased the incidence of cells at interphase with multiple nuclei (Figure 2C), as well as those at mitotic phase with abnormal chromosome segregation and multiple centrosomes (Figure 2D). The numbers of cells with abnormal multi-nuclei or abnormal centrosome segregation were counted (*n* = 300 cells, *p* < 0.0001; Figures 2C and 2D).

To ascertain the dysregulation of centrosomes, we further monitored the effect of GSK3β inhibition on centrosomes in HCT116 cells by live-imaging analysis of cells expressing green fluorescent protein (GFP)-centrin 2 (another known centrosome marker) fusion protein. Following treatment with AR-A014418 and SB-216763 at 25 µM, a significant number of GFP-centrin 2 repetitively moved forth and back until cells underwent mitotic death (Supplementary Movie 1 [control], S2 [AR-A014418], S3 [SB-216763], and S4 [GSK3β RNAi]). These findings allow us to hypothesize that GSK3β participates in the mitotic process by interacting with the dynein-TPR mitotic mediator complex at centrosomes. Immunoprecipitation of whole extracts from HCT116 cells synchronized at the M-phase with anti-γ-tubulin antibody resulted in co-precipitation of GSK3β, TPR, and dynein (Figure 2F, left panels). Consistent with immunostaining and live-cell imaging data (Figures 2A, 2B, and Supplementary Movies 5–8), treatment with either GSK3β inhibitor decreased co-immunoprecipitation of TPR and dynein with γ-tubulin (Figure 2F, middle and right panels). These results suggest that GSK3β colocalizes and potentially works together with TPR and dynein in centrosomes during mitosis.

### GSK3β inhibition reduced TPR expression and altered TPR-dynein centrosomal localization in CRC cells

**Figure 3:**
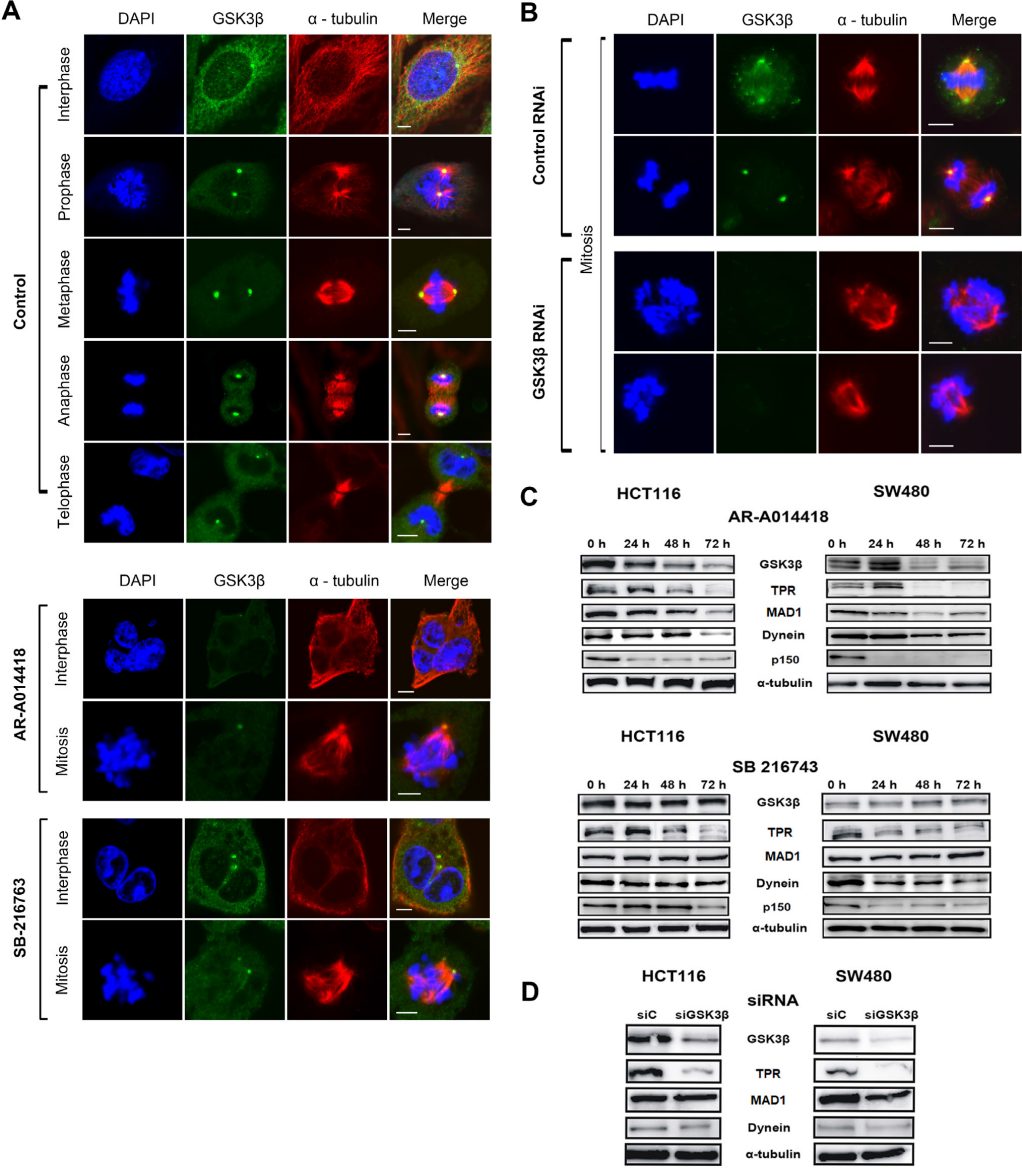
GSK3β inhibition induced aberrant spindle polarity and altered expression of TPR and dynein in CRC cells HCT116 cells were immunostained with anti-α-tubulin (spindle apparatus marker; red) and anti-GSK3β (green) antibodies, counterstained with DAPI (chromatin; blue) and examined by confocal microscopy. (**A**) Representative images of HCT116 cells in the process of mitosis in control group and groups treated with 25 μM of AR-A014418 or SB-216763. (**B**) Comparison of images of mitotic HCT116 cells transfected with control or GSK3β-specific siRNA for 72 hours. (A, B) Scale bar = 5 µm. (**C**) Expression of GSK3β, TPR, MAD1, p150, and dynein in HCT116 (left panels) and SW480 (right panels) cells treated with 25 μM AR-A014418 or SB-216763 for indicated periods. (**D**) Comparison of immunoblotting indicating levels of expression of GSK3β, TPR, and its associated proteins (MAD1 and dynein) in HCT116 (left panels) and SW480 (right panels) cells transfected with control (siCTL) or GSK3β-specific siRNA (siGSK3β) for 72 hours.

**Figure 4:**
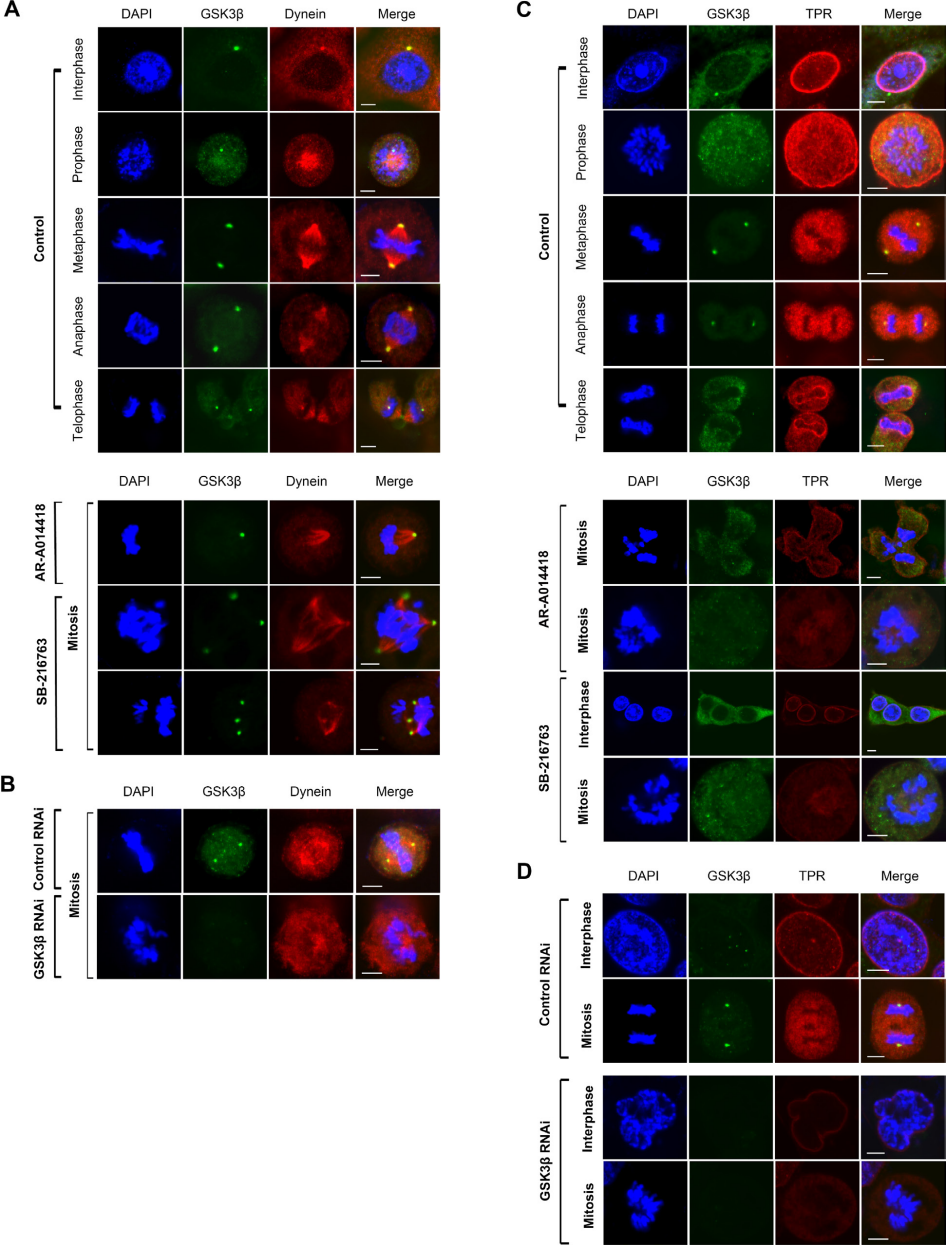
GSK3β inhibition abolished dynein and TPR centrosomal localization, and caused aneuploidy in cell division HCT116 cells were immunostained with anti-dynein (red) (**A, B**) or anti-TPR (red) (**C, D**), and anti-GSK3β (green) (A–D) antibodies and examined by confocal laser microscopy. Chromatin was counter-stained with DAPI (blue). Representative images of mitotic HCT116 cells treated with DMSO (control) or 25 μM of AR-A014418 or SB-216763 (A, C), or transfected with non-specific (control RNAi) or GSK3β-specific (GSK3β RNAi) siRNA (B, D). (A–D) Scale bar = 5 µm.

TPR interacts with spindle checkpoints throughout the cell cycle. A previous report found that TPR overexpression enhanced multinucleation and aneuploidy formation. TPR also recruits checkpoint proteins to the dynein complex along spindles during mitosis [44]. We hypothesized that mitotic abnormalities were also caused by disruption of TPR-dynein axis function after GSK3β inhibition. To examine spindle polarity defects, we co-immunostained GSK3β with the spindle marker α-tubulin. As predicted, treatment with either GSK3β inhibitor or a GSK3β-specific siRNA significantly enhanced spindle defects compared with the respective controls (Figure 3A and 3B, and Supplementary Figure 4). A previous study reported that GSK3β phosphorylates dynein and alters its motility via a reduction of Ndel1 binding to intermediate chains [18]. Therefore, the results shown above (spindle defects) prompted us to investigate whether GSK3β affects TPR and dynein [44] in CRC cells during mitosis. To examine TPR and dynein protein expression after GSK3β inhibition, we performed immunoblotting analysis over a time course (from 0 to 72 h) with GSK3β inhibitors and GSK3β-specific siRNA, respectively. Notably, TPR and dynein were temporally reduced in both HCT116 and SW480 cells; however, expression of the TPR binding partner MAD1 and dynein binding partner p150 protein were not in synchrony with regard to individual inhibitor or cell line (Figure 3C). TPR expression was decreased when GSK3β expression was depleted in HCT116 and SW480 cells (Figure 3D and Supplementary Figure 5). Consistent with immunoblotting data, observation with confocal laser microscopy revealed that inhibition of GSK3β activity induced the mislocalization of dynein and TPR in both HCT116 and SW480 cells (Figure 4, and Supplementary Figures 6 and 7).

**Figure 5:**
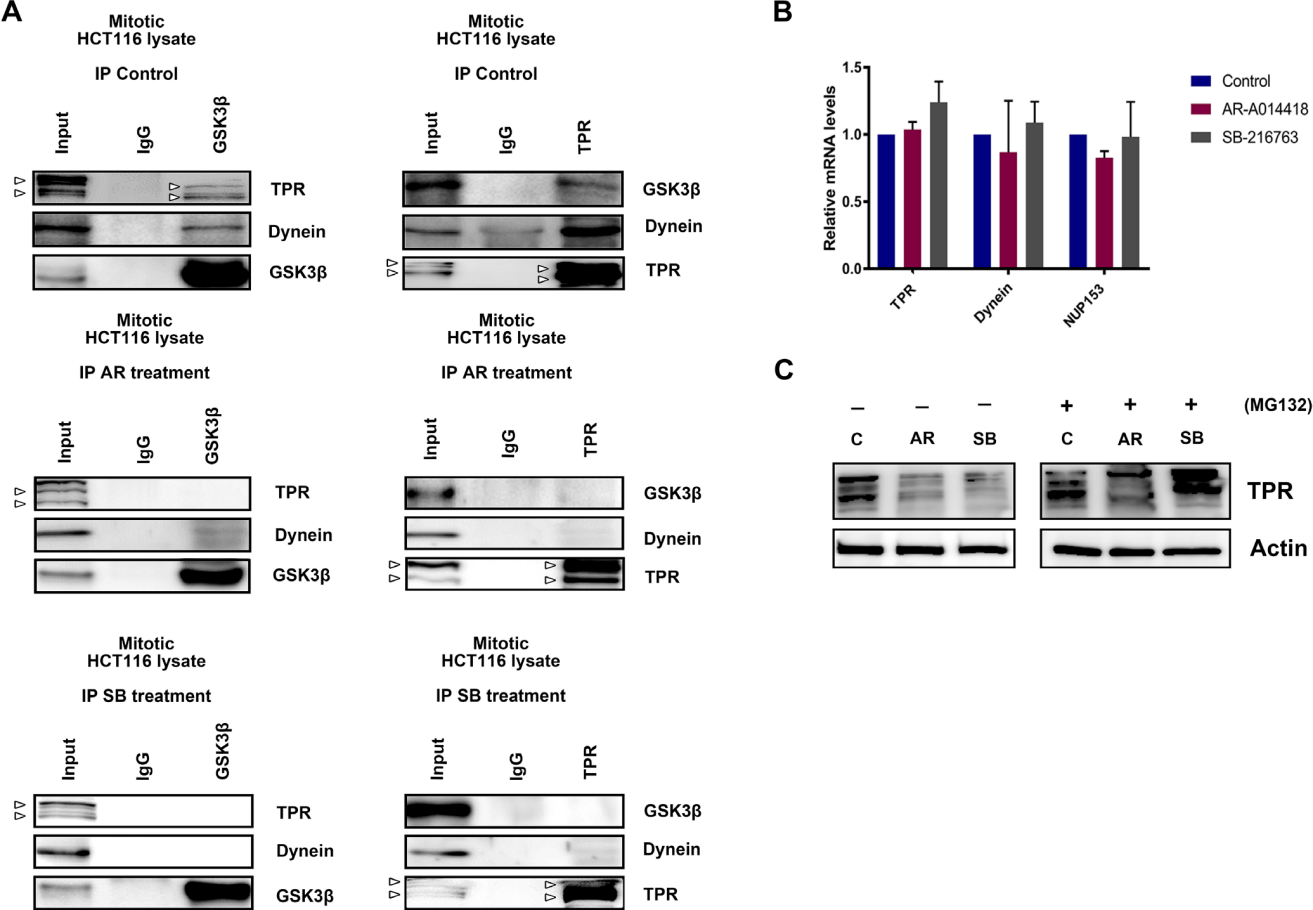
GSK3β inhibition induced TPR mislocalization, leading to its proteasomal degradation (**A**) HCT116 cells were treated with DMSO (control), AR-A014418 (AR), or SB-216763 (SB), and then synchronized at mitotic phase as shown in Figure 2E. Immunoprecipitates (IP) from extracts of mitotic HCT116 cells with anti-GSK3β (left panels) and anti-TPR (right panels) antibodies, and nonspecific rabbit IgG, were analyzed by immunoblotting with indicated antibodies (arrowheads indicate TPR and its isoform). (**B**) qRT-PCR analysis of *TPR*, *DYNEIN*, and *NUP153* mRNA in HCT116 cells after treatment with DMSO or GSK3β inhibitors (AR-A014418 and SB-216763) for 48 hours. Data show mean ± SD of three separate experiments. (**C**) Expression of *TPR* in HCT116 cells treated with DMSO (C), 25 μM AR-A014418 (AR), or SB-216763 (SB) for 72 hours in the presence or absence of MG132 (10 μM) for 8 hours.

Next, we explored whether GSK3β biochemically interacts with dynein and TPR during the process of mitosis. Immunoprecipitation of extract from HCT116 cells synchronized at the M-phase with an antibody against GSK3β co-precipitated dynein and TPR (Figure 5A, left panels), while an antibody against TPR co-precipitated GSK3β and dynein (Figure 5A, right panels). Treatment of cells with GSK3β inhibitors attenuated the biochemical association among GSK3β, dynein, and TPR (Figure 5A, middle and lower sets of panels). We further investigated the signaling cascade position between TPR and GSK3β. As predicted, both TPR and pGSK3β^Y216^ (active form) localization to the centrosome were remarkably reduced after GSK3β inhibition (Supplementary Figure 8A). In addition, by using a constitutively active form of GSK3β (GSK3β S9F-HA), we found that the number of cells with multi-nuclei and abnormal chromosome segregation was decreased in HCT116 cells transfected with GSK3β S9F-HA compared with cells transfected with empty vector following treatment with GSK3β inhibitors (Supplementary Figures 8B and 8C). The effects of GSK3β S9F-HA transfection were associated with partial recovery of TPR expression (Supplementary Figure 8D). Together, these results suggest an important role of active GSK3β in the centrosome during mitosis. Furthermore, we found that the depletion of *TPR* did not alter the expression or phosphorylation of GSK3β (Supplementary Figure 9), suggesting that TPR functions downstream of GSK3β. To further confirm that TPR reduction and mislocalization resulted from its ubiquitin-mediated proteasomal degradation, we performed qRT-PCR to measure the level of *TPR* mRNA and immunoblotting to measure the level of TPR protein following treatment with GSK3β inhibitors in the absence and presence of MG132, a proteasome inhibitor. As predicted, we detected no significant changes in levels of *TPR* mRNA (Figure 5B), but observed the recovery of TPR expression in cells treated with GSK3β inhibitors in the presence of MG132 (Figure 5C right panels). This result indicates that GSK3β inhibition destabilizes TPR by inducing its proteasomal degradation. Together, these morphological and biochemical findings suggest that GSK3β participates in proper localization and function of the mitotic mediators dynein and TPR to sustain mitosis for colon cancer cell propagation.

### Expression and localization of TPR in human CRC tissues and effect of GSK3β inhibition on SW480 cell xenografts in mice

**Figure 6:**
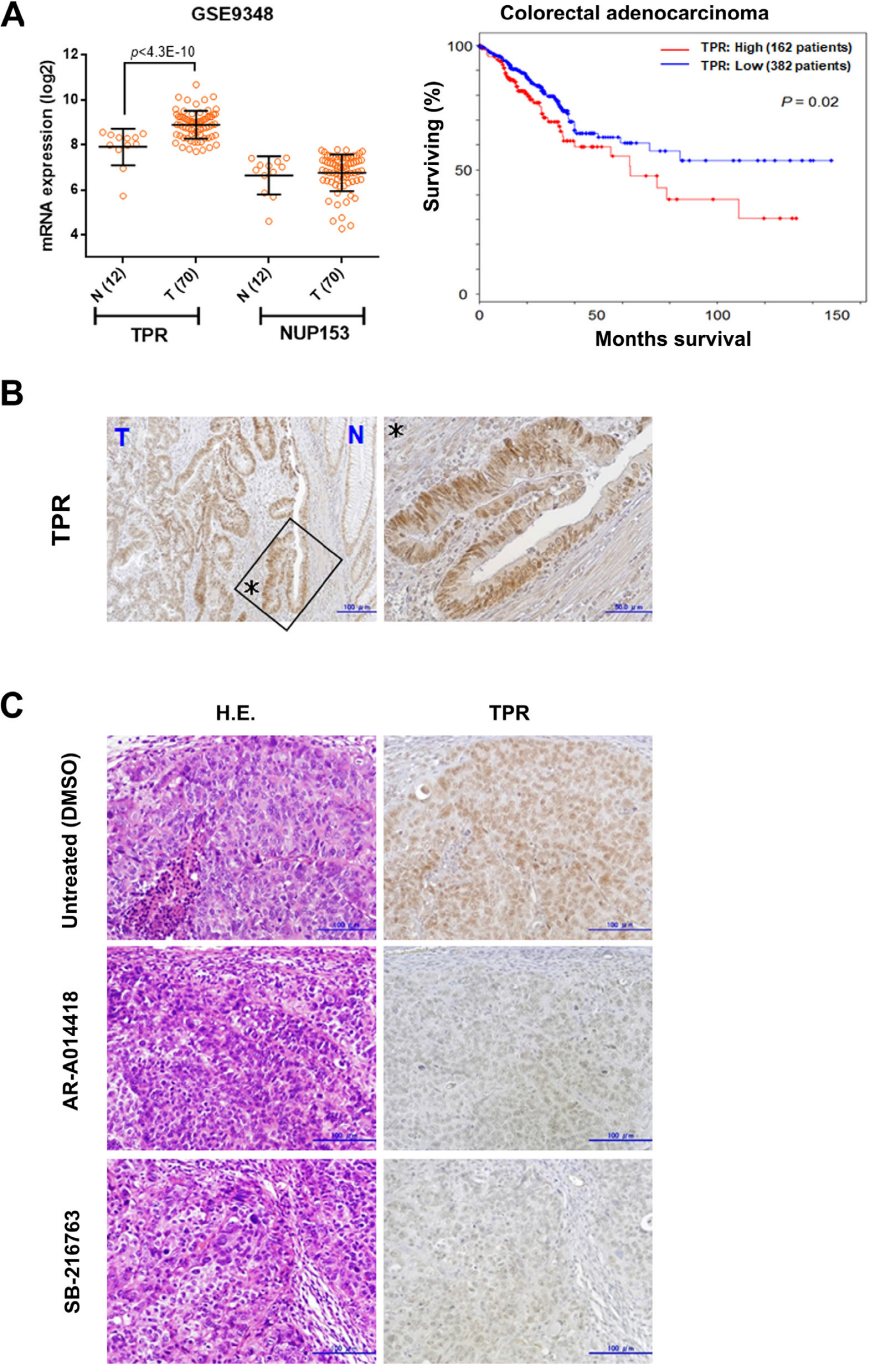
Nucleoporin TPR is upregulated in colon cancers (**A**) *TPR* mRNA expression across normal (N) and tumor (T) tissues of colon cancer patients examined from GEO (series GSE9348). Data show mean ± SD (left panel). High *TPR* expression (mRNA expression z-Scores (RNA Seq V2 RSEM)³ mean + 0.1 SD) was associated with poor disease-free survival of colorectal adenocarcinoma patients in TCGA cohorts (right panel). (**B**) Expression and subcellular localization of TPR in the primary tumor of a colon cancer patient. Asterisks (enlarged region) indicated pattern of TPR nuclear rim staining occurred exclusively in most cancer cells. (**C**) Immunohistochemical findings of expression and localization of TPR in SW480 xenograft tumors in mice treated with DMSO (control), AR-A014418, or SB-216763 (both 5 mg/kg body weight) for 5 weeks. Scale bar = 100 μm; H.E., hematoxylin and eosin.

Finally, to validate our cellular working model (Supplementary Figure 10) in CRC patient samples, we examined *TPR* expression in human CRC tumors. Primary samples from ExpO/gene expression profile/eGWASs were collected from public repositories and analyzed [48]. Evaluation of gene expression profiles in CRC identified an upregulation of *TPR*, but not *NUP153* (another nuclear basket protein that binds TPR) [26], in CRC patient databases (*p* < 0.001; Figure 6A left panel). This is consistent with the result shown in Figure 5C, as well as our previous studies showing higher expression and activity of GSK3β in tumors compared with normal tissues in CRC patients [12, 15]. Kaplan-Meier analysis of TCGA cohorts revealed that *TPR* overexpression was significantly correlated with poorer outcome of colorectal adenocarcinoma patients (Log-rank test, *P* = 0.02; Figure 6A right panel). Immunohistochemical examination of primary tumors from 20 CRC patients (Supplementary Table 3) showed higher expression of TPR in nuclei and nuclear membranes in cancer cells compared with non-neoplastic crypt cells (Figure 6B).

We previously demonstrated the therapeutic effect of GSK3β inhibitors (AR-A014418 and SB-216763) against SW480 and HT-29 colon cancer cell xenografts in athymic mice [14, 15]. Upon examining SW480 xenograft tumors by immunohistochemistry, we found lower levels of TPR expression in the tumors of mice treated with AR-A014418 and SB-216763 compared with sham (DMSO)-treated mice (Figure 6C).

## DISCUSSION

The effect of GSK3β inhibition on survival and proliferation of CRC cells observed in this study was associated with cell cycle arrest at G2/M phase and the induction of apoptosis. As previously reported [16–18, 22], our morphological and biochemical analyses showed that GSK3β transiently localized and bound to the components of centrosomes and mitotic spindles, the key functional microstructures driving mitosis, during mitosis of CRC cells. Although the efficiency of the two GSK3β inhibitors (AR-A014418 and SB-216763) on several cellular processes was different, these inhibitors exerted the same pharmacological effect impairing the mitotic process of the respective colon cancer cell lines. Consequently, our study indicate that induction of mitotic catastrophe underpins the cancer therapeutic effect of GSK3β inhibition. Suppression of cell survival by GSK3β inhibition was consistent with our previous studies showing induction of morphologically apoptotic bodies/cells, terminal deoxynucleotidyl transferase dUTP (2’-deoxyuridine 5’-triphosphate) nick end labeling (TUNEL)-positive cells, and ladder-like DNA fragmentation, and the suppression of human telomerase reverse transcriptase and telomerase-mediated cell immortality pathways in colon cancer cells [12–15].

Efficiency of the two GSK3β inhibitors and GSK3β-specific siRNA was variable among the colon cancer cells examined in this study. Observed differences could be attributable to differences in the sensitivities or susceptibilities of the respective cancer cells to the same inhibitors and/or GSK3β-specific siRNA. The biochemical IC_50_ concentrations of AR-A014418 and SB-216763 (reported to be 27 nM and 9 nM, respectively) were determined by *in vitro* kinase assays using purified enzyme and specific substrate peptides [46, 47]. These studies on the development and characterization of the above-mentioned GSK3β inhibitors reported the range of pharmacological dosages used in the present and previous studies [11–15], as well as in other studies (reviewed in Ref. No. 7–9). The primary mechanism of action for the two pharmacological inhibitors is to block the ATP-binding domain of GSK3β, but not to directly change phosphorylation of its S9 or Y216 residues. Importantly, we previously demonstrated that these inhibitors directly inhibit the activity (ability) of GSK3β to phosphorylate its substrate in colon cancer SW480 and HCT116 cells, which are also used in the present study, and that this effect is dosage-dependent in the reported pharmacological dosage range [13, 15]. Therefore, the effects of these inhibitors on cells at dosages used in our present and previous studies [11–15] were based on their pharmacological effect against cellular GSK3β activity, but not by off-target effects against other kinases or side effects. However, it is important to confirm the therapeutic effects of these two inhibitors are specific for GSK3β activity, but not non-specific or off-target effects. To achieve this, we tested a GSK3β-specific siRNA on colon cancer cells. In many cases, the effect of an enzyme largely depends on its biological or catalytic activity rather than its amount. Such is the case for GSK3β and we previously reported that the pharmacological GSK3β inhibitors (AR-A014418 and SB-216763) inactivate GSK3β in cancer cells within an hour after treatment, while the GSK3β-specific siRNA takes longer than 48 hours to efficiently, but not completely, deplete GSK3β expression in the same cells [13, 15]. Therefore, we believe that pharmacological inhibitors more quickly inhibit GSK3β activity in cells, leading to a stronger biological effect on colon cancer cells than treatment with a GSK3β-specific siRNA, as shown in Figure 1F and Supplementary Figure 2.

In close relation to the present study, our research group recently reported the results of a clinical trial for treatment of recurrent glioblastoma patients with repurposed GSK3β-inhibiting medicines in combination with temozolomide [49]. In this clinical study, we observed that GSK3β-inhibiting medicines attenuated cell proliferation and invasion of patients’ brain tumors, as well as primary tumors in a mouse glioblastoma model. This clinical study suggests that GSK3β inhibition may also attenuate cell viability and proliferation in primary colon cancer cells. Therefore, investigation of the effects of GSK3β inhibition on primary CRC cells (e.g. primary cultures of tumors from CRC patients and patient-derived tumor xenografts) will facilitate therapeutic application in CRC patients.

We previously found an unexpected role of NUP358/Ran-binding protein 2 showing that, in addition to its primary function as a structural component of NPC in cancer cells division, it is also involved in mitotic catastrophe in HeLa cells [32]. Subsequently, a recent study confirmed its role in mitosis in CRC cells [33], and proposed the precision treatment of CRC by repurposing vinorelbine, a microtubule-targeting agent approved for several cancer types other than CRC. In addition to this Nup, we have demonstrated that TPR, a nuclear pore basket constituent present in cells at interphase, facilitates mitotic processes in cancer cells [43, 44]. To explore the putative molecular mechanism controlling TPR and dynein complexes in cellular mitosis, we hereby showed the unique finding that mitotic aberration (or catastrophe) induced by GSK3β inhibition coincided with impaired interaction among TPR, dynein, and GSK3β in mitotic CRC cells.

Impairment of the control of cell cycle is one of the characteristic hallmarks of cancer [50]. This notion has prompted the development of cancer therapy targeting mitosis, which is central to the cell cycle program [3–5, 51]. Early generations of clinically evaluated cancer therapeutics included agents targeting microtubule dynamics and inhibitors of mitotic kinases such as cyclin-dependent kinases (CDKs) [51], aurora kinases [52] and polo-like kinases [53, 54]. Despite numerous clinical trials, the efficacy of these agents has been disappointing due to their narrow therapeutic windows in association with substantial adverse effects affecting bone marrow, nerves, and gastrointestinal tract tissue [reviewed in 3]. However, recent evidence supporting the preferential dependence of cancer cells on interphase CDKs, including CDK4 and CDK6, suggests higher efficiency of a new class of agents targeting CDKs [55, 56]. We previously demonstrated that GSK3β sustains expression of CDK4, CDK6, and cyclin D1, which results in phosphorylation-dependent inactivation of RB cell cycle regulator, in CRC and pancreatic cancer cells [15, 57]. Therefore, the disruption of interphase cell cycle procession would also underlie the cancer therapeutic effect of GSK3β inhibition.

While genomic/chromosomal instability is recognized as the global genomic hallmark of cancer [50], excessive instability renders cancer cells intolerable to survival or propagation [58, 59]. Therefore, to relief unfavorable effects of the mitotic kinase inhibitors discussed above, genomic/chromosomal instability has emerged as the attractive cancer therapeutic target in cell cycle and mitotic process [reviewed in 3]. Based on this knowledge, mitotic mediators controlling centrosome biodynamics and mitotic spindle assembly (two critical processes for accurate chromosomal alignment that ensure genome stability) have become potential targets in cancer therapy [60]. As chromosomal instability is the representative consequence of aberration in mitotic processes, our present study suggests that the disturbance of chromosomal segregation, mitotic spindle assembly, and centrosome duplication in CRC cells following treatment with GSK3β inhibitors may cause excessive chromosomal instability that is compatible with the therapeutic effect of GSK3β inhibition.

Our immunofluorescence and live-cell imaging results provide the first evidence that GSK3β inhibition disrupted TPR and dynein forming a sub-complex in centrosomes and facilitated aberrant multi-centrosomes formation. Our study thus raises a new notion that TPR and dynein interact and participate in promotion of mitotic process in CRC cells under the control by GSK3β (Supplementary Figure 10). Indeed, GSK3β phosphorylates TPR at S2059 *in silico* [45]. We speculate that GSK3β phosphorylation regulates the stability of TPR and the transient interaction between TPR and its mitotic binding partners such as MAD1, dynein, or Aurora A during mitosis (Supplementary Figure 10). Further, our work also provides new insights into mechanisms for cancer cell mitosis, and reinforces this strategy for cancer treatments targeting GSK3β [7]. It is still unclear exactly how GSK3β is recruited to the centrosome/spindle pole, but this mechanism seems to involve a cascade of events and potentially involves dynein motors.

Finally, we examined the expression of *TPR* in human CRC tumors from the database. Primary samples from ExpO/gene expression profile/eGWASs were collected from public repositories [48]. As predicted, evaluation of gene expression profiles in CRC identified an upregulation of *TPR* in GEO database (*P* < 0.001, Figure 6A). Collectively, this study sheds light on a novel mitotic function of GSK3β in the regulation of TPR-dynein mediated centrosome homeostasis, which may facilitate the discovery of new treatment regimens for cancer targeting mitosis. Future clinical investigations should explore how GSK3β-TPR contributes to carcinogenesis.

## MATERIALS AND METHODS

### Cell culture

Human colon cancer HCT116, SW480, LoVo, and HT-29 cells were obtained from American Type Culture Collection. Cells were propagated in Dulbecco’s Modified Eagle’s Medium (DMEM) supplemented with 10% (v/v) fetal bovine serum (Life Technologies) and 50 U/mL penicillin-streptomycin (Nacalai Tesque). Cells were cultured in a humidified incubator at 37° C with 5% CO_2_. Cells were synchronized in S-phase by thymidine block using 2 mM thymidine (Figure 2E) [43, 44].

### Cell viability assay

Colon cancer cells were seeded in 96-well culture plates at a density of 3,000 cells per well. Cells were then treated with DMSO or GSK3β inhibitors (AR-A014418 [46] or SB-216763 [47]) (Sigma-Aldrich) at the indicated concentrations. At each time point (0, 12, 24, 48, and 72 hours), cell viability was determined using a WST-8 assay kit (Cell Counting Kit-8, Dojindo Laboratories). Optical density was measured using a microplate reader (Model 680, BIO-RAD) at 450 nm, and the results are shown as the mean of optical density and standard deviations of each four-well set.

### Plasmid, RNA interference, and treatment with GSK3β inhibitors

The expression plasmid for constitutively active GSK3β tagged with an HA epitope (pCI-GSK3β S9F-HA) was constructed by inserting a GSK3β cDNA fragment amplified using a set of primers (Supplementary Table 1) into the pCI vector (Promega), and then performing an amino acid substitution of the S9 residue to phenylalanine (F) by mutagenesis with a set of primers listed in Supplementary Table 1. Small interfering (si)RNA duplexes targeting GSK3β (5′-AUCUAGCUUUCUCAUGAUCUGGAGC-3′), TPR-specific siRNA (sc-45343), and control siRNA (sc-37007) were purchased from Santa Cruz Biotechnology. Plasmid and siRNA were transfected using Lipofectamine 2000 (Invitrogen) according to the manufacturer’s protocol [43, 44]. HCT116 and SW480 cells were plated onto 12- or 6-well tissue culture plates at a density of 1 × 10^5^ cells per well. Cells were transfected with siRNA and observed 72 hours after transfection. To examine the effect of inhibition of GSK3β activity, cells were treated with 25 µM AR-A014418 [46] or 25 µM SB-216763 [47] (Sigma-Aldrich) and collected at the indicated periods.

### Immunocytochemistry, confocal microscopy, and live-cell imaging

This study used antibodies against GSK3β (32391, Abcam) and TPR (sc-101294, Santa Cruz Biotechnology). Monoclonal antibodies against dynein (D5167, clone 74.1), α-tubulin (DM1A), and γ-tubulin were from Sigma-Aldrich. Secondary antibodies (Alexa Fluor- or Rhodamine-conjugated) were from Molecular Probes (Life Technologies).

For observation by confocal microscopy, M-phase–synchronized HCT116 cells on coverslips were washed in phosphate-buffered saline (PBS) and fixed for 10 min in 4% paraformaldehyde in PBS. Cells were then permeabilized with 0.3% Triton X-100 in PBS for 10 minutes at room temperature. Cells were then incubated with each of the indicated primary antibodies for 3 hours, washed three times, and incubated with Alexa Fluor-conjugated secondary antibody for 2 hours. After washing with PBS, cells were incubated with the second primary antibody for 3 hours, followed by incubation with Rhodamine-conjugated secondary antibody for 2 hours for double immunostaining. Cells were mounted onto coverslips with ProLong Gold Antifade reagent with 4′,6-diamidino-2-phenylindole (DAPI; Invitrogen).

Real-time live-cell imaging was performed using HCT116 GFP-centrin and SW480 GFP-TPR stable cells treated with DMSO, GSK3β inhibitor, or GSK3β siRNA. Images were acquired 24 and 48 hours after treatment using an Olympus FV10i-LIV laser-scanning confocal microscope with a 60X PlanApo/1.45NA DIC objective.

### Immunoprecipitation and Western blotting

Procedures used for immunoprecipitation and Western blotting were described previously [32, 43]. Briefly, mitotic HCT116 cells were collected, washed with PBS, and lysed in 1 mL of cold lysis buffer containing protease inhibitor mixture. Lysates were centrifuged for 30 minutes at 4° C at 14,000 × *g*. The resultant supernatants were pre-cleared with 50 µL of protein A/G beads slurry (Santa Cruz Biotechnology), mixed with 10 µL of various antibodies (as specified), and incubated for 2 hours at 4° C with rocking. Beads were then washed five times with 500 µL of lysis buffer. After the last wash, 50 µL of 1× sodium dodecyl sulfate polyacrylamide gel electrophoresis (SDS-PAGE) blue loading buffer (New England Biolabs) was added to the bead pellet and heated for 10 minutes at 95° C before loading. Signals were detected with an enhanced chemiluminescence system (GE Healthcare,) and quantified using a LAS-4000 image analyzer (Fuji Film) according to the manufacturer’s specifications.

For conventional Western blotting analysis of cells at various conditions, we used the same antibodies described above for immunofluorescence staining and immunoprecipitation. In addition, we also used antibodies against GSK3α and β (05-412, Millipore), α-dynactin p150 subunit (612709, BD Biosciences), cyclin B1 (4138P, Cell Signaling), histone H3 (H3; 07-690, Millipore), H3 phosphorylated in its S10 residue (p-H3^S10^; 06-570, Upstate), PARP1 (sc-7150, Santa Cruz), MAD1 (sc-47746, Santa Cruz), β-actin (sc-47778, Santa Cruz), Y216-phosphorylated GSK3β (pGSK3β^Y216^; 612312, BD Biosciences), S9-phosphorylated pGSK3β^S9^ (9336, Cell Signaling), and HA-tag (AM1008a, Abgent).

### Cell cycle profile analysis

Fluorescence-activated cell sorting (FACS) analysis was performed as described previously [32, 43]. Briefly, 72 hours after treatment with DMSO, 25 μM AR-A014418, or 25 μM SB-216763, HCT116 cells were trypsinized, washed twice with PBS, and fixed in 70% ethanol at –20° C overnight. Fixed cells were resuspended in PBS containing 50 µg/mL RNase A (Nacalai Tesque) and 50 µg/mL propidium iodide (PI) (Sigma-Aldrich). Cellular DNA content was analyzed using a FACS Canto II with FACS Diva software (BD Bioscience).

### cDNA preparation and quantitative real-time RT-PCR assay

RNA was prepared using a NucleoSpin RNA isolation kit (Macherey–Nagel), and cDNA was synthesized using the ThermoScript RT-PCR system (Takara). Quantitative real time RT-PCR was performed using a Thermal Cycler Dice Real Time System with SYBR Premix Ex Taq II (Takara). Relative mRNA expression levels of target genes were calculated using glyceraldehyde-3-phosphate dehydrogenase (*GAPDH*) as an internal control. Primers are listed in Supplementary Table 2.

### Patients and tissue specimens

We analyzed the primary tumor tissues of 20 CRC patients undergoing surgery at our University Hospital (Supplementary Table 3). Written informed consent was obtained from each patient in accordance with institutional guidelines. Our investigation was conducted in accordance with ethical standards and the Declaration of Helsinki, and according to national and international guidelines, after approval by our Institutional Review Board.

### Human CRC xenograft tumors in mice treated with GSK3β inhibitor

In our previous study, we demonstrated the efficacy of GSK3β inhibitors (AR-A014418 and SB-216763) against SW480 cell xenografts in athymic mice [14]. For histological and immunohistochemical examination, representative sections were prepared from archived tissue blocks of formalin-fixed and paraffin-embedded tumors of rodents treated with DMSO (control), AR-A014418 (5 mg/kg body weight), or SB-216763 (5 mg/kg body weight) for 5 weeks.

### Histological and immunohistochemical examination

Respective paraffin sections of CRC tumors and xenografts were histologically examined by hematoxylin and eosin staining. Expression of TPR in tumor tissues was immunohistochemically examined using an avidin-biotin-peroxidase complex method described in our previous studies [15]. Representative paraffin sections placed on silanized slides (Dako) were treated by microwaving in citrate buffer to unmask antigens, incubation with 0.3% H_2_O_2_ in methanol, and subsequent incubation with 10% normal goat serum to block non-specific immunohistochemical reactions. Pretreated tissue sections were incubated with a rabbit polyclonal antibody against TPR (diluted 1:100; Santa Cruz). After incubation with the antibody, sections were incubated with biotinylated goat anti-rabbit IgG (Vector Laboratories) diluted 1:200 in PBS containing 10% normal goat serum. For mouse xenograft tumors, final concentrations of 1% bovine serum albumin and 10% normal mouse serum (DakoCytomation) were added to the diluent of anti-rabbit IgG to prevent cross-reaction with endogenous mouse IgG [14, 15].

### Bioinformatics and data analysis

*TPR* mRNA expression in CRC was obtained from GEO (Accession No. GSE9348, https://www.ncbi.nlm.nih.gov/geo/GSE9348). Survival curves from The Cancer Genome Atlas (TCGA, http://cancergenome.nih.gov/) were obtained through cBio Cancer Genomics Portal (http://www.cbioportal.org/). All bioinformatics data were accessed and analyzed accordingly [61] .

### Statistical analysis

Statistical analysis was performed using GraphPad PRISM 7. Data are shown as means ± standard deviation (SD). Comparisons between respective groups were determined using an unpaired *T*-test or two-way ANOVA. *P* values < 0.05 were considered statistically significant.

## SUPPLEMENTARY MATERIALS FIGURES, TABLES AND VIDEOS



















## Abbreviations

CDK(s): cyclin-dependent kinase(s)
CRC: colorectal cancer
DAPI: 4’,6-diamidino-2-phenylindole
DMSO: dimethyl sulfoxide
FACS: fluorescence-activated cell sorting
GAPDH: glyceraldehyde-3-phosphate dehydrogenase
GFP: green fluorescent protein
GEO: gene expression omnibus
GSK3β: glycogen synthase kinase-3β
NPC: nuclear pore complex
Nup(s): nucleoporin(s)
PARP-1: poly [ADP-ribose] polymerase 1
PBS: phosphate-buffered saline
PI: propidium iodide
RNAi: RNA interference
RT-PCR: reverse transcription-PCR
S9: serine 9
siRNA: small interfering RNA
TCGA: the Cancer Genome Atlas
TPR: translocated promoter region
TUNEL: terminal deoxynucleotidyl transferase dUTP (2’-deoxyuridine 5’-triphosphate) nick end labeling
Y216: tyrosine 216
