# Supplementary material for: Colorectal cancer cells require glycogen synthase kinase-3β for sustaining mitosis via translocated promoter region (TPR)-dynein interaction
Source: Oncotarget. 2018 Jan 30;9(17):13337–52. doi: 10.18632/oncotarget.24344 (PMC5862582; doi:10.18632/oncotarget.24344)
Supplement: Supplementary file 1 [file oncotarget-09-13337-s001.pdf]

Colorectal cancer cells require glycogen synthase kinase-3β for sustaining mitosis via translocated promoter region (TPR)-dynein interaction

SUPPLEMENTARY MATERIALS

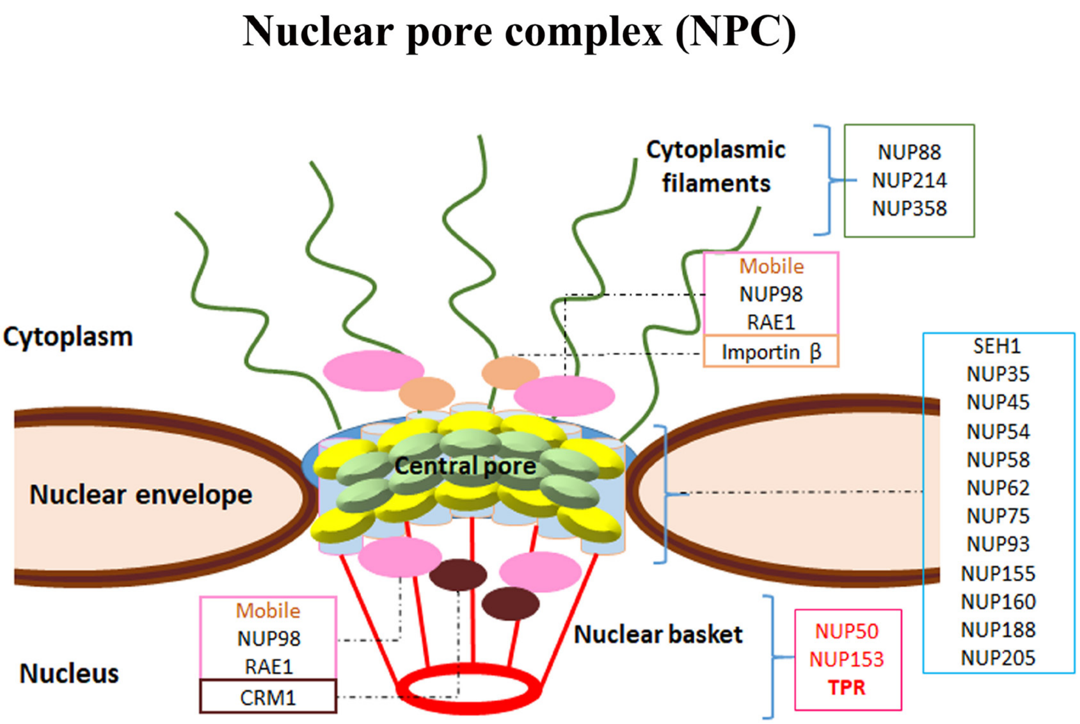

Supplementary Figure 1: Schematic representation of mammalian nuclear pore and nuclear pore complex (NPC). NPC is composed of cytoplasmic filaments (NUP358/Ran-binding protein 2, NUP214, and NUP88; green) that extend into cytoplasm; a central pore that consists of nucleoporin SEH1, NUP35, NUP45, NUP54, NUP58, NUP62, NUP75, NUP93, NUP155, NUP160, NUP188, and NUP205; and a nuclear basket (NUP50, NUP153 and TPR; red) that extends into nucleoplasm. Nucleoporin TPR forms a bridge between NPC and underlying chromatin, binds directly to NUP153 and plays a role in exportation of nuclear proteins and RNA. It is implicated in multiple additional cellular functions and assigned multiple functions, including being as a chromatin scaffolding element that facilitates transcriptional telomeric chromatin organization by establishing perinuclear heterochromatin exclusion zones. It is also involved in SUMOylation, mitotic spindle bipolarity, centrosome formation, and the control of cellular senescence [43, 44].

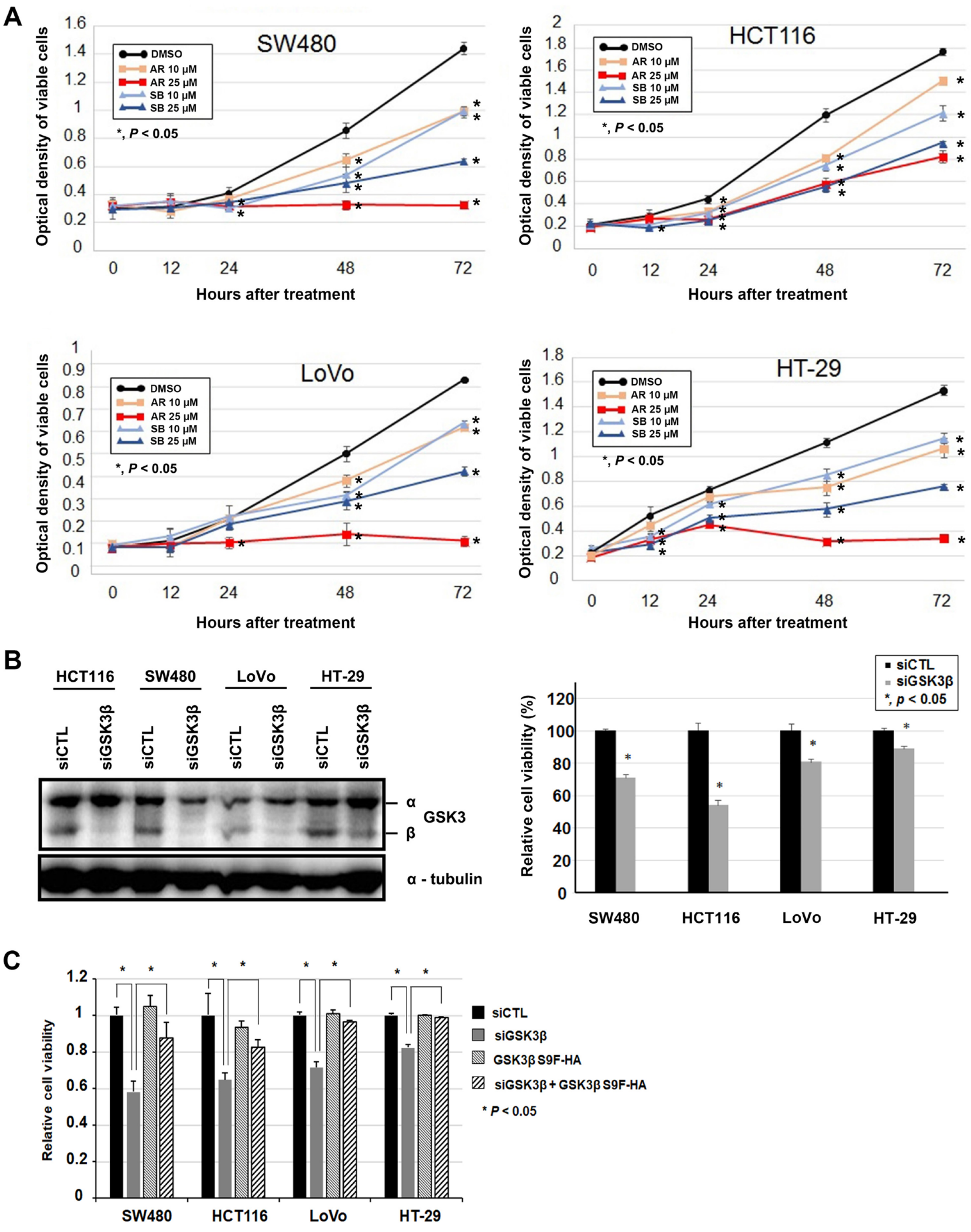

Supplementary Figure 2: Effects of GSK3β inhibition on the survival and proliferation of colon cancer cells. (**A**) Effect of GSK3β inhibitors on the survival of colon cancer SW480, HCT116, LoVo and HT-29 cells. Relative cells viability was determined after treatment with DMSO, AR-A014418 and SB-216763 at two different dosages (10 μM and 25 μM) for 72 hours. Viable cells at indicated time points were determined by using WST-8 assay kit. Data indicate means ± SD of four separate experiments. *P *value* < 0.05, statistically significant difference between the cells treated with DMSO and either of AR-A014418 or SB-216763. (**B**) Immunoblotting analysis for expression of GSK3β in colon cancer cells transfected with non-specific (siCTL) and GSK3β-specific siRNA (siGSK3β). Expression of α-tubulin was monitored as a loading control (left panels). Effects of GSK3β RNAi on the viability of colon cancer cell lines. Relative numbers of viable cells were determined 72 hours after transfection with siCTL or siGSK3β (right panel). Asterisks, statistically significant difference between cells transfected with siCTL or siGSK3β. (**C**) Relative numbers of viable cells were determined 72 hours after transfection with siCTL, siGSK3β, plasmid containing constitutively active mutant form of GSK3β (GSK3β S9F-HA), respectively alone, or with siGSK3β and GSK3β S9F-HA in combination to reconfirm that viability/proliferation of the respective cancer cell lines depends on GSK3β expression. All assays were performed in triplicates, and data indicate means ± SDs. Asterisks, statistically significant difference between the data.

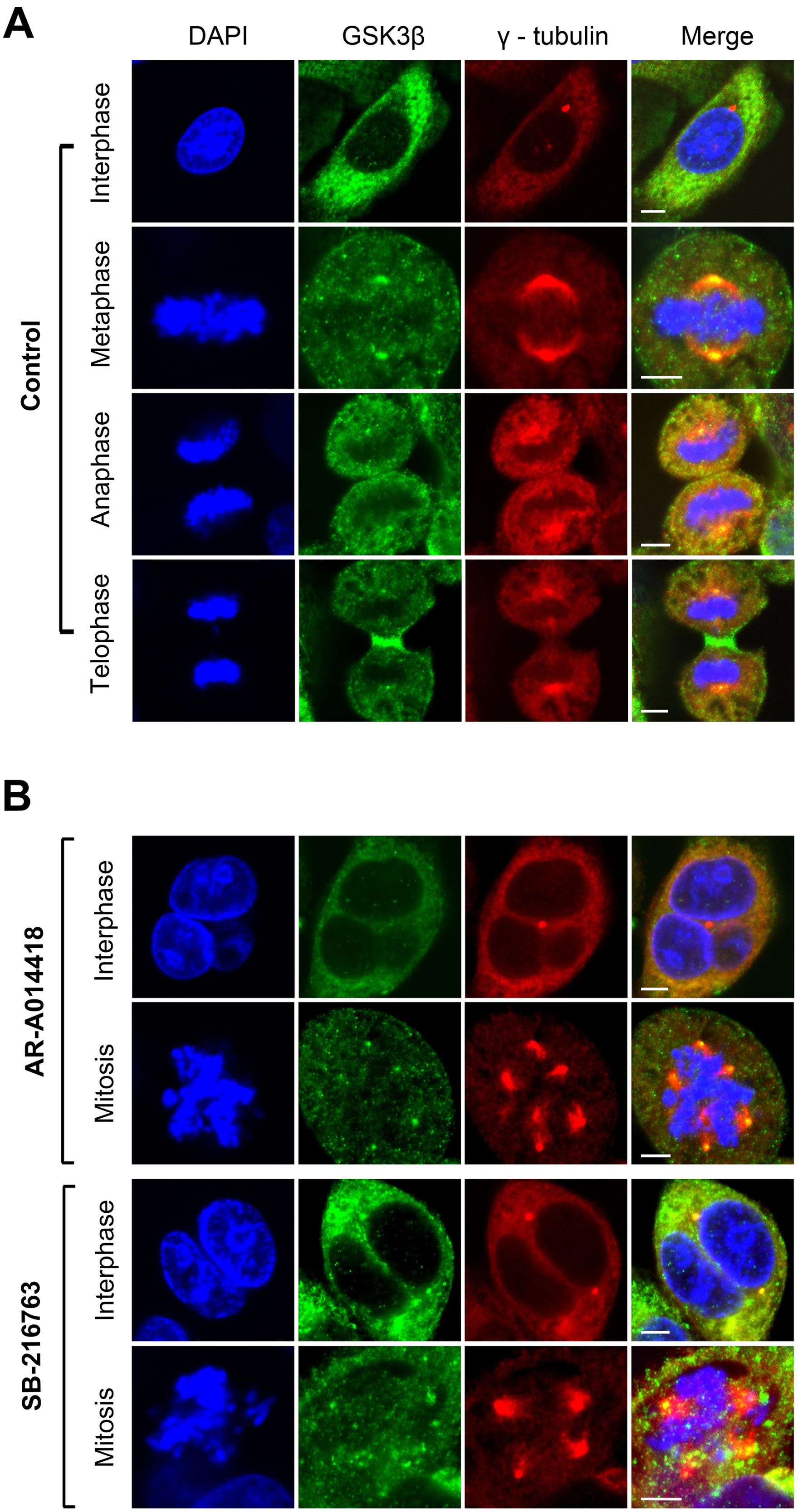

Supplementary Figure 3: GSK3β inhibition induced abnormal centrosome number in mitotic SW480 cells. (**A**) Representative images of mitotic SW480 cells treated with DMSO, or (**B**) 25 μM of AR-A014418 or SB-216763, immunostained with anti-γ-tubulin (red) and anti-GSK3β (green) antibodies and examined by confocal microscopy. Chromatin was counter-stained with DAPI (blue). Scale bars = 5 μm. [Related to Figure 3 for HCT116 cells].

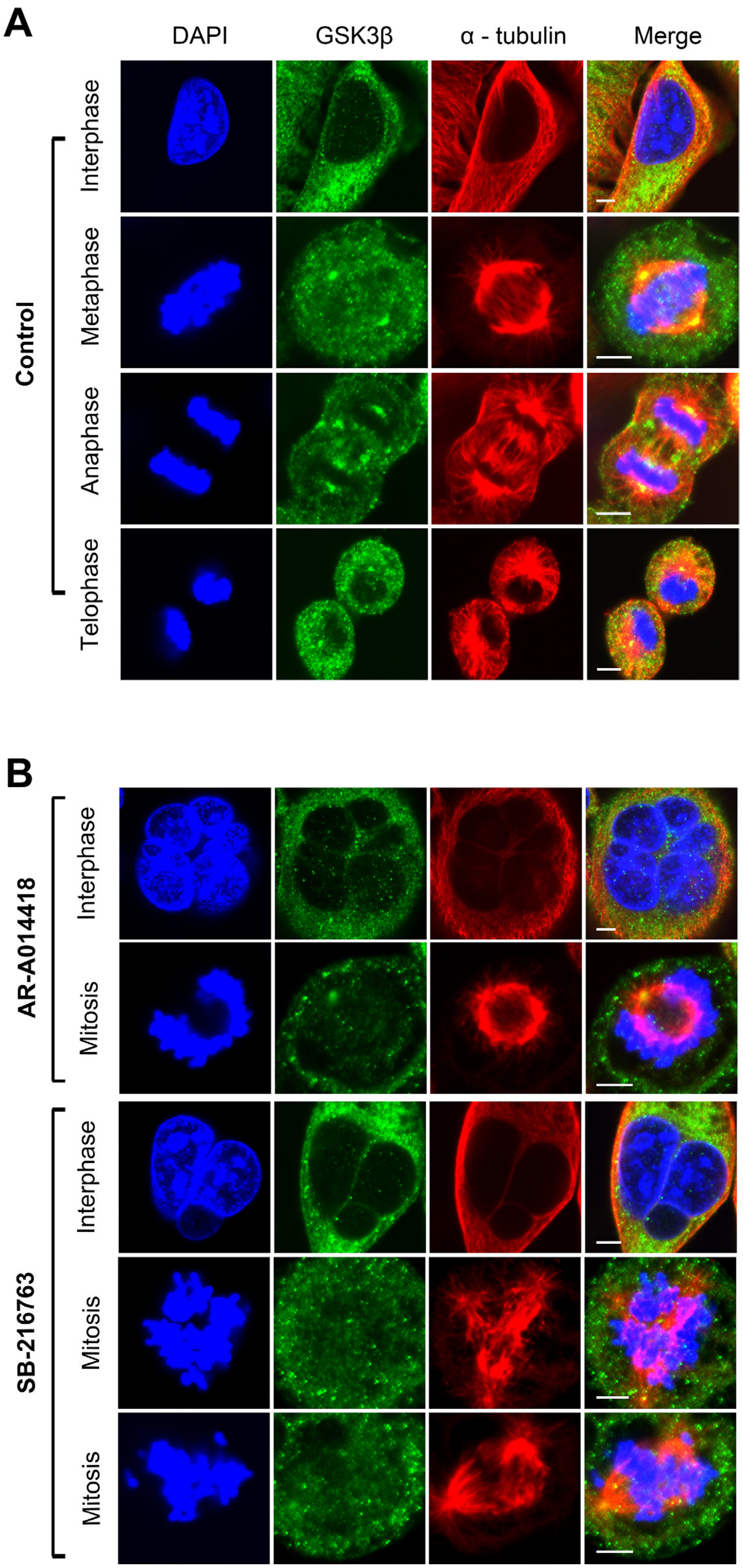

Supplementary Figure 4: GSK3β inhibition abolished spindle bi-polarity and caused multi-nuclei and abnormal chromosome segregation in mitotic SW480 cells. (**A**) Representative images of mitotic SW480 cells treated with DMSO, or (**B**) 25 μM of AR-A014418 or SB-216763, immunostained with anti-α-tubulin (red) and anti-GSK3β (green) antibodies and examined by confocal microscopy. Chromatin was counter-stained with DAPI (blue). Scale bars = 5 μm. [Related to Figure 3 and 4 for HCT116 cells].

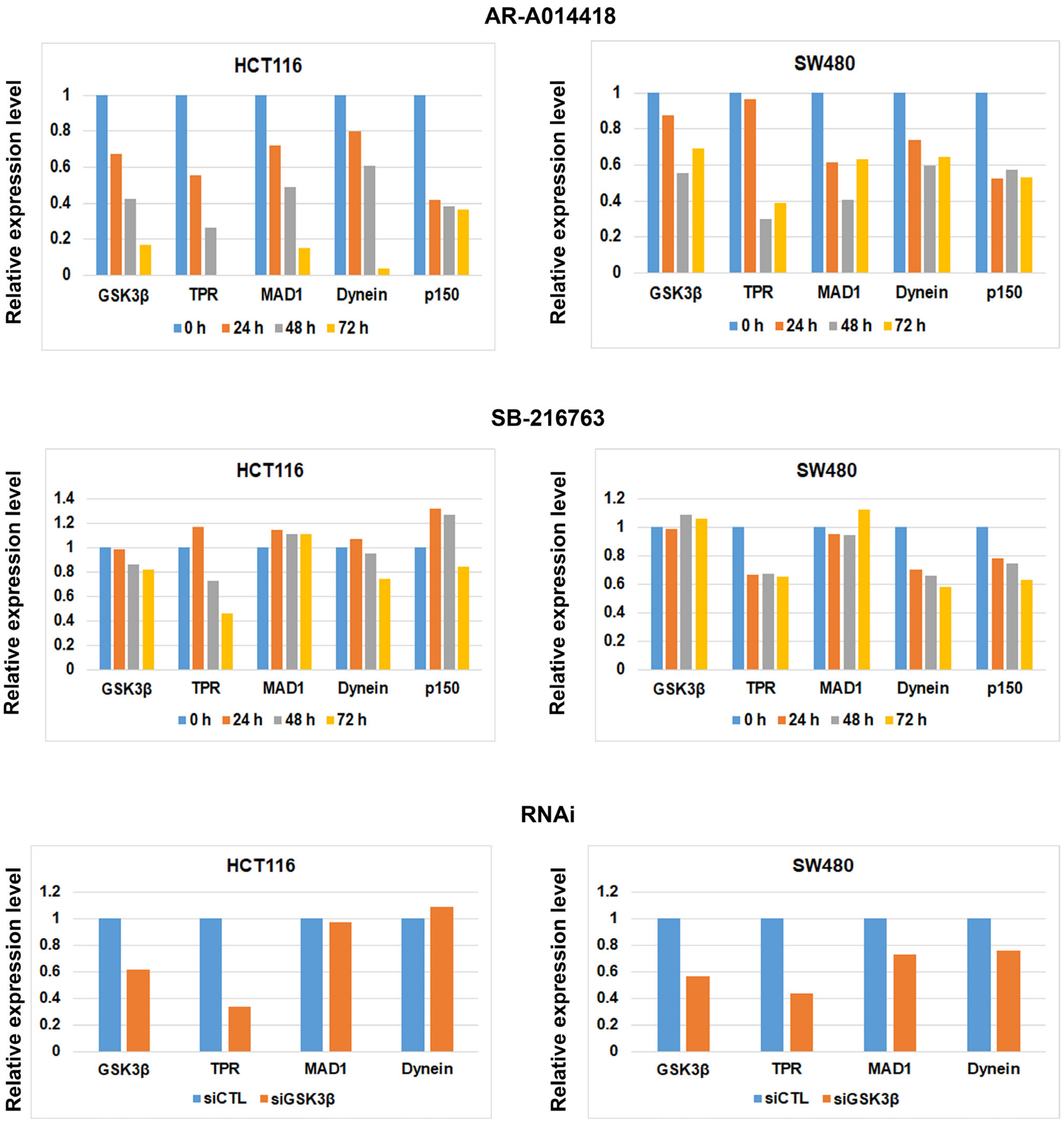

Supplementary Figure 5: Quantification of the effects of GSK3β inhibition on the expression of TPR and dynein in CRC cells. Intensity of the respective signals in the Western blotting results shown in Figure 3C and 3D was measured using a densitometry. Relative intensity of GSK3β, TPR, MAD1, Dynein and p150 were calculated by normalizing to α-tubulin intensity as an internal control and to the signals in control (0 hr in Figure 3C) and non-specific siRNA (siCTL in Figure 3D).

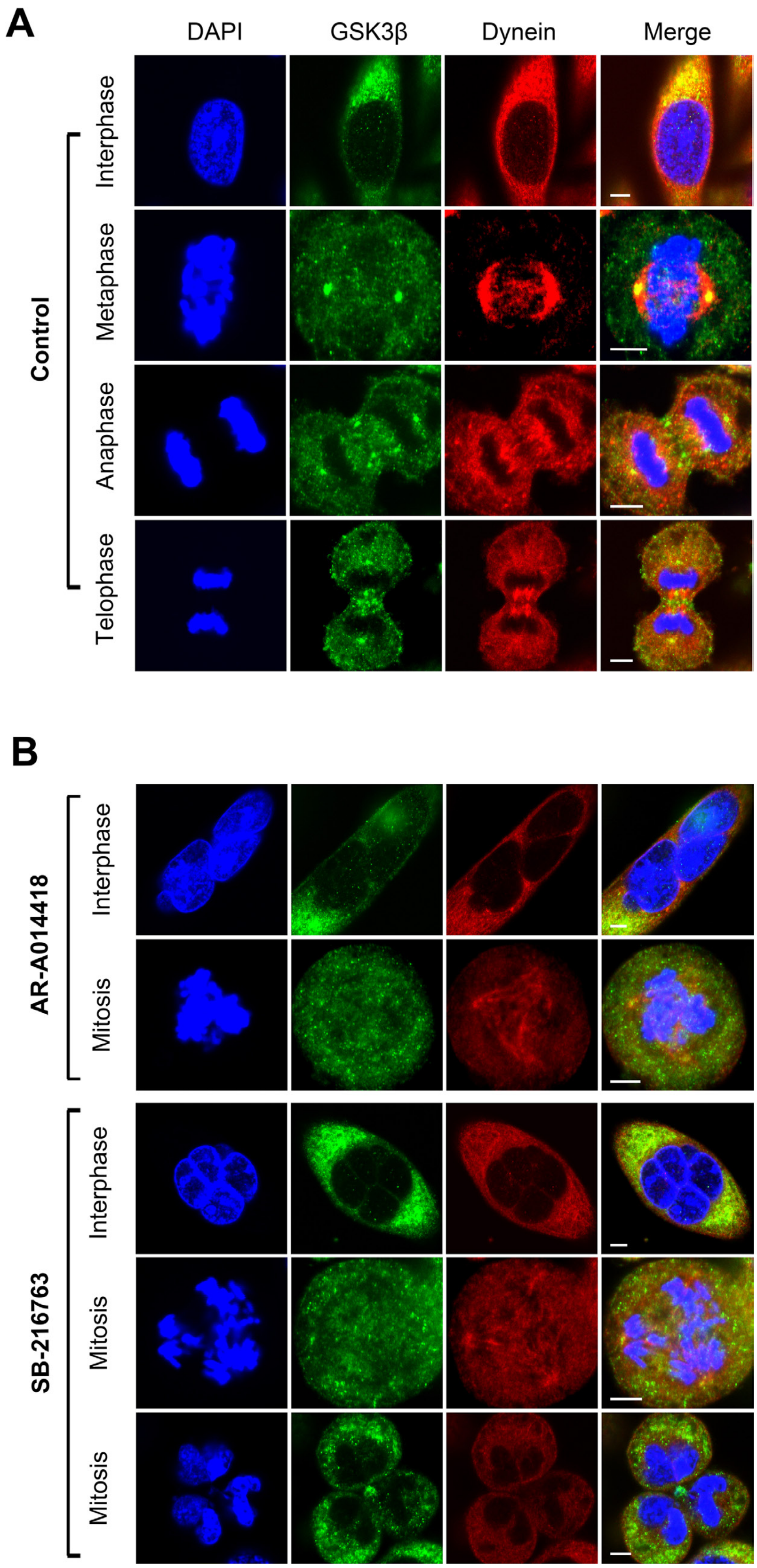

Supplementary Figure 6: GSK3β inhibition abolished dynein localization to centrosomes and caused aneuploidy in mitotic SW480 cells. (**A**) Representative images of mitotic SW480 cells at different mitotic phases treated with DMSO, or (**B**) 25 µM of AR-A014418 or SB-216763, immunostained with anti-dynein (red) and anti-GSK3β (green) antibodies and examined by confocal microscopy. Chromatin was counter-stained with DAPI (blue). Scale bars = 5 µm. [Related to Figure 5 for HCT116 cells].

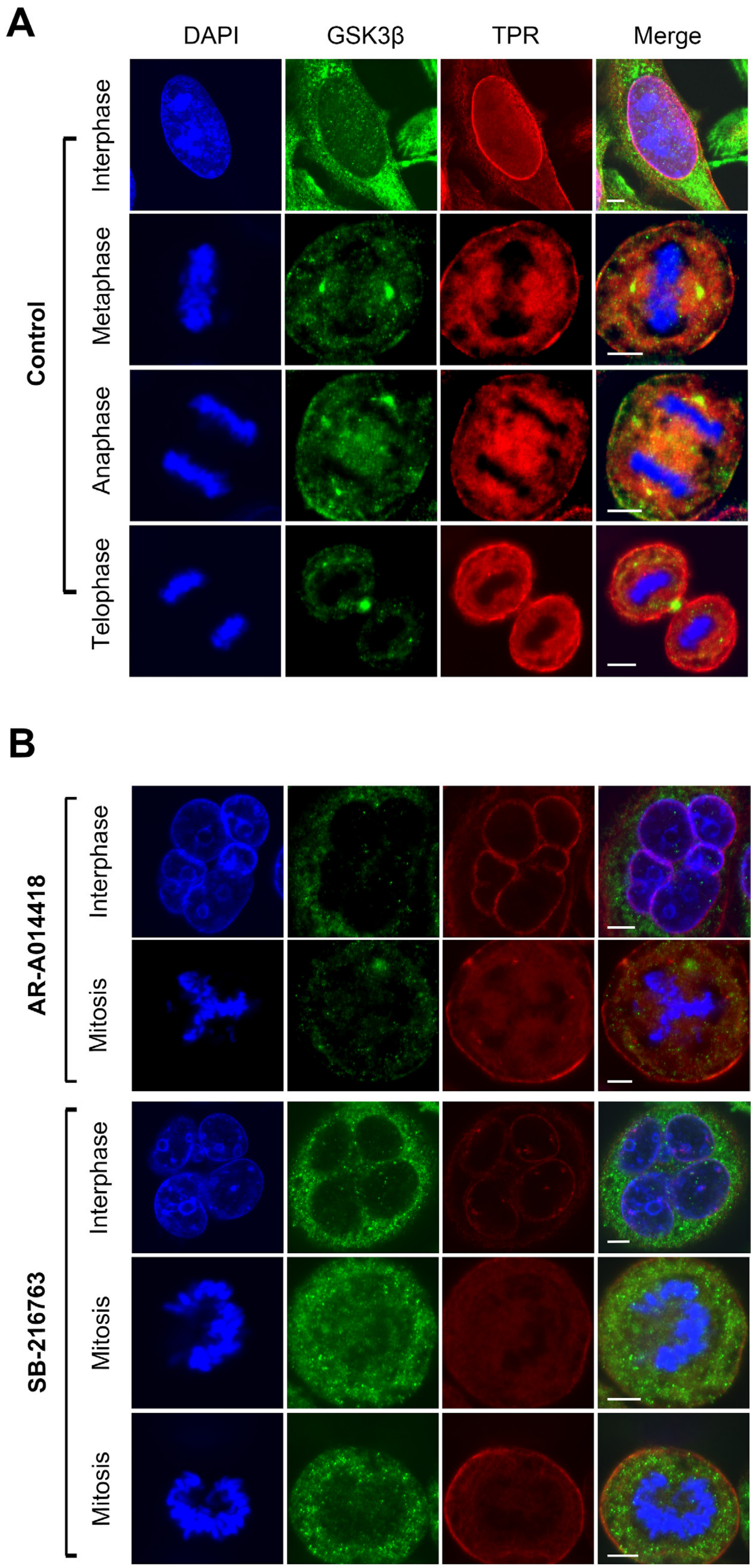

Supplementary Figure 7: GSK3β inhibition abolished TPR localization to centrosomes and caused aneuploidy in mitotic SW480 cells. (**A**) Representative images of mitotic SW480 cells at different mitotic phases treated with DMSO, or (**B**) 25 µM of AR-A014418 or SB-216763, immunostained with anti-TPR (red) and anti-GSK3β (green) antibodies and examined by confocal microscopy. Chromatin was counter-stained with DAPI (blue). Scale bars = 5 µm. [Related to Figure 5 for HCT116 cells].

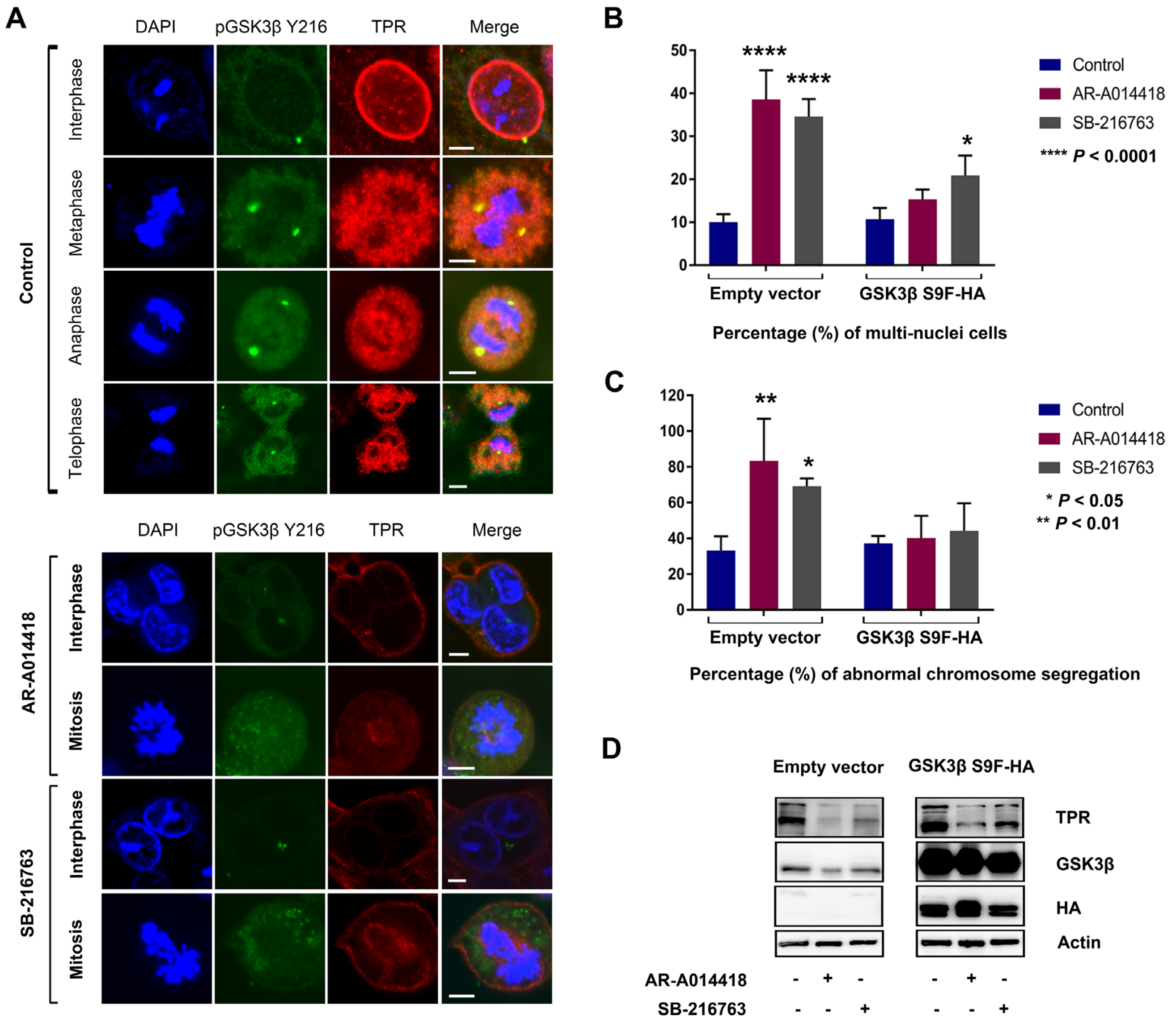

Supplementary Figure 8: Active GSK3β has an important role for proper chromosome segregation during mitosis in HCT116 cells. (**A**) Representative images of mitotic HCT116 cells at different mitotic phases treated with DMSO, 25 μM of AR-A014418 or SB-216763, immunostained with anti-TPR (red) and anti-pGSK3β^{Y216} (green) antibodies and examined by confocal microscopy. Chromatin was counter-stained with DAPI (blue). Scale bars = 5 μm. (**B**) Percentage of multi-nuclei and (**C**) abnormal chromosome segregation in HCT116 cells transfected with empty vector or GSK3β S9F-HA in the presence or absence of GSK3β inhibitor. (**D**) Immunoblot of TPR, GSK3β and HA in HCT116 cells transfected with empty vector or GSK3β S9F-HA for 72 h and detected with indicated antibodies. Expression of β-actin was monitored as a loading control.

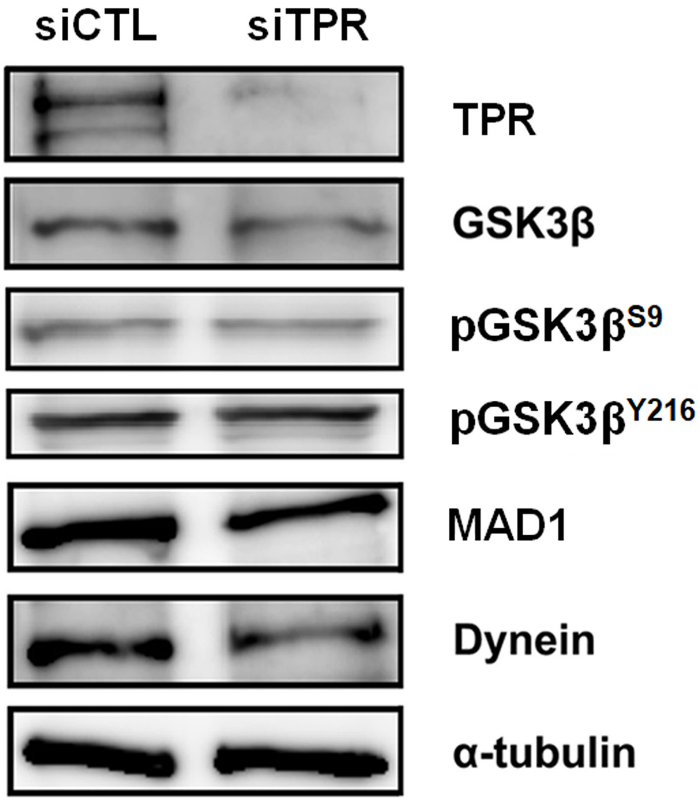

Supplementary Figure 9: Effects of TPR depletion on the expression of GSK3β, MAD1 and dynein and on the phosphorylation of GSK3β in colon cancer cells. HCT116 cells were transfected with either non-specific (siCTL) or TPR-specific siRNA (siTPR) for 72 hours. Expression of TPR, GSK3β, MAD1, dynein and phosphorylation of S9 (pGSK3β^{S9}) and Y216 (pGSK3β^{Y216}) residues in GSK3β were examined by Western blotting. Expression of α-tubulin was monitored as a loading control.

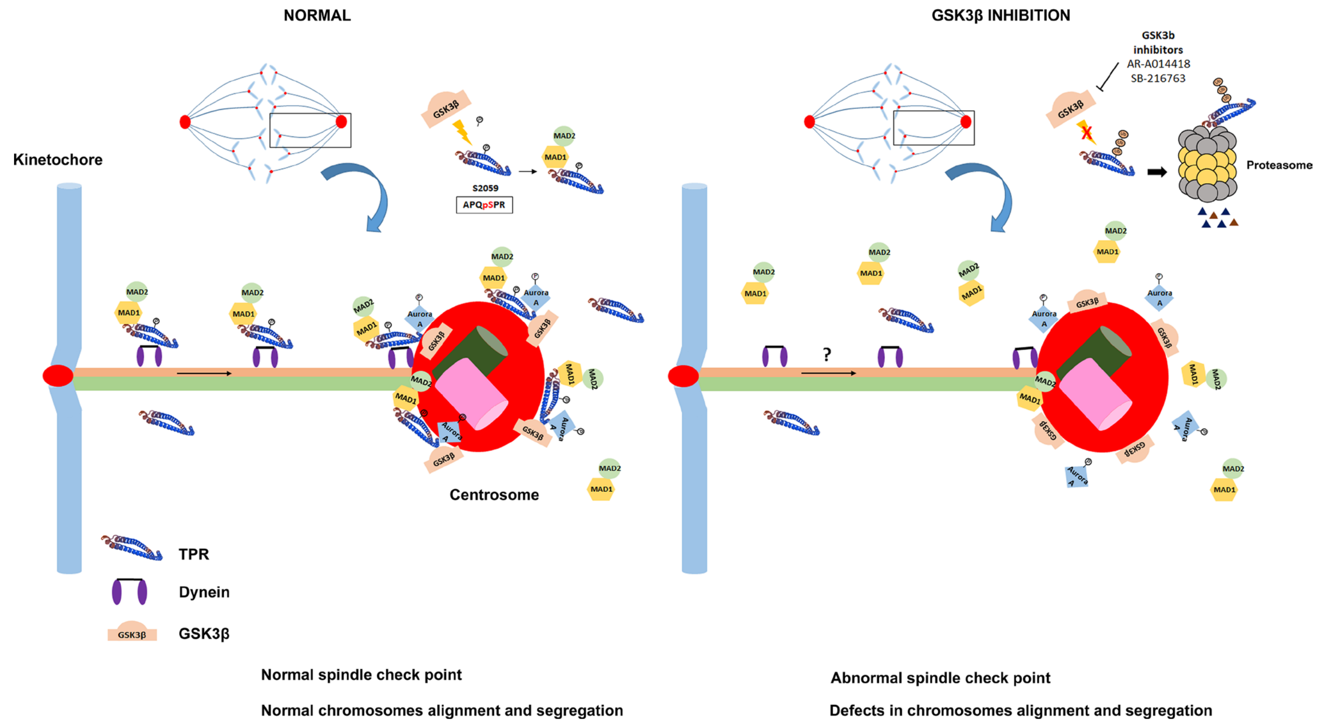

Supplementary Figure 10: A mechanistic model of interaction between GSK3β and TPR-dynein complex in mitosis. GSK3β phosphorylates TPR or dynein that localizes along the spindle poles/centrosomes to facilitate TPR and dynein activity at the centrosomes. GSK3β inhibition disrupts TPR-dynein centrosomal localization, enhances formation of multiple centrosomes, multi-nuclei and aneuploidy. GSK3β inhibition also destabilizes TPR via its ubiquitination and proteasomal degradation. Localization of GSK3β in centrosomes is critical for proper spindle check-point and anaphase entry.

Supplementary Table 1: Primers to generate GSK3β S9F-HA plasmid

Target	Forward	Reverse
GSK3β	5′- ATAGAATTCATGTCAGGGC GGCCCAGAACC -3′	5′- TATAGTCGACTCAAGCGTAAT CTGGAACATCGTATGGGTAGG TGGAGTTGGAAGCTGATG -3′
S9F	5′- CAGAACCACCTTCTTTGCGGAG -3′	5′- CTCCGCAAAGAAGGTGGTTCTG -3′

Abbreviations; GSK3β, glycogen synthase kinase-3β; S9F, serine 9 phenylalanine.

Supplementary Table 2: qRT-PCR primers list

Target	Forward	Reverse
DIC	5′- AGCTGAGCTAGAGCGCAAAA -3′	5′- GCAAAGCCTCTGTCTCTCGT -3′
TPR	5′- AGTTGGGACCACCAGTTCAG -3′	5′- TGCCACCTATTCCTGGAGTC -3′
Nup153	5′- GCAGCTCTGCCTTTGGTAAC -3′	5′- AAGACAAATGGGGTGACAGC -3′
GAPDH	5′- GTCAGTGGTGGACCTGACCT -3′	5′- AGGGGTCTACATGGCAACTG -3′

Abbreviations: DIC, dynein intermediate chain; TPR, translocated promoter region; GAPDH, glyceraldehyde 3-phosphate dehydrogenase.

Supplementary Table 3: Clinical characteristics and pathology of 20 colorectal cancer patients

No.	Age/Gender	Tumor site	Tumor histology	TNM stage
1	49/F	sigmoid colon	MD	T3N2M0, stage IIIb
2	48/M	rectum	Muc	T3N2M1, stage IV
3	72/F	cecum	MD	T3N1M0, stage IIIb
4	80/M	sigmoid colon	MD	T3N0M0, stage IIa
5	85/F	ascending colon	MD	T3N0M0, stage IIa
6	62/M	cecum	Muc	T3N2M1, stage IV
7	87/F	ascending colon	PD > MD	T4aN0M0, stage IIb
8	72/F	ascending colon	Muc > WD	T2N0M0, stage I
9	60/M	rectum	MD	T3N1M0, stage IIIb
10	50/F	rectum	MD	T3N0M0, stage IIa
11	69/M	rectum	MD	T3N0M2, stage IV
12	45/M	descending colon	WD	T2N0M0, stage I
13	63/F	ascending colon	MD	T3N0M0, stage IIa
14	81/F	transverse colon	PD	T3N0M0, stage IIa
15	56/F	rectum	WD	T3N1M0, stage IIIb
16	75/F	ascending colon	MD > PD	T3N2M0, stage IIIb
17	76/M	ascending colon	MD	T3N0M0, stage IIa
18	57/M	rectum	WD	T1N0M0, stage I
19	51/F	sigmoid colon	MD	T3N0M0, stage IIa
20	61/F	sigmoid colon	MD	T3N0M0, stage IIa

Abbreviations; F, female; M, male; MD, moderately differentiated adenocarcinoma; Muc, mucinous adenocarcinoma; PD, poorly differentiated adenocarcinoma; WD, well differentiated adenocarcinoma.
Tumor histology and TNM stage according to the Union for International Cancer Control (UICC) Classification.

Supplementary Movie 1: Live imaging of mock-treated HCT116 cells expressing GFP-centrin 2 (time interval, 16 hr; display rate, 1 frame/3 min). See Supplementary_Movie_1

Supplementary Movie 2: Live imaging of 25 µM AR-A014418-treated HCT116 cells expressing GFP-centrin 2 (time interval, 16 hr; display rate, 1 frame/3 min). See Supplementary_Movie_2

Supplementary Movie 3: Live imaging of 25 µM SB-216763-treated HCT116 cells expressing GFP-centrin 2 (time interval, 16 hr; display rate, 1 frame/3 min). See Supplementary_Movie_3

Supplementary Movie 4: Live imaging of GSK3β-specific siRNA-treated HCT116 cells expressing GFP-centrin 2 (time interval, 16 hr; display rate, 1 frame/3 min). See Supplementary_Movie_4

Supplementary Movie 5: Live imaging of mock-treated SW480 cells expressing GFP-TPR (time interval, 16 hr; display rate, 1 frame/3 min). See Supplementary_Movie_5

Supplementary Movie 6: Live imaging of 25 µM AR-A014418-treated SW480 cells expressing GFP-TPR (time interval, 16 hr; display rate, 1 frame/3 min). See Supplementary_Movie_6

Supplementary Movie 7: Live imaging of a 25 µM of SB-216763-treated SW480 cell expressing GFP-TPR (time interval, 16 hr; display rate, 1 frame/3 min). See Supplementary_Movie_7

Supplementary Movie 8: Live imaging of a GSK3β-specific siRNA-treated SW480 cell expressing GFP-TPR (time interval, 16 hr; display rate, 1 frame/3 min). See Supplementary_Movie_8
